# Function and mechanism of GBP1 in the development and progression of cervical cancer

**DOI:** 10.1186/s12967-023-04837-6

**Published:** 2024-01-02

**Authors:** Senyu Wang, Yajing Zhang, Xiumin Ma, Yangchun Feng

**Affiliations:** Clinical Laboratory Center, Cancer Hospital Affiliated to Xinjiang Medical University, Xinjiang, China

**Keywords:** Guanylate binding protein 1 (GBP1), Tumor immunity, Cervical cancer, Alternative splicing (AS), Cancer promotion

## Abstract

Guanylate binding protein 1 (GBP1) is the most concerned member of the GBP family, which has a series of effects such as anti-infection and anti-angiogenesis. Its role in malignant tumors including cervical cancer is still controversial. We aim to explore the effects of GBP1 on cervical cancer through bioinformatics and related experiments. In this study, we first found that GBP1 was generally expressed in cervical cancer in various online databases and was closely related to immune invasion. Secondly, we used multicolor immunofluorescence technology to verify the expression of GBP1 in cervical cancer tissues and its relationship with immune invasion, and explored its relationship with the prognosis of patients with cervical cancer. Knockdown and overexpression assays of GBP1 in vitro were used to prove GBP1 as a potential oncogene of cervical cancer, and its carcinogenicity was verified by in vivo experiment. In order to explore the potential mechanism of GBP1 in promoting cancer, RNA-seq was performed on GBP1 overexpression and knockdown expression cell lines, and GBP1 knockdown and overexpression were found to be associated with many RNA alternative splicing events, suggesting that GBP1 maybe a RNA binding protein (RBP) which affect the biological characteristics of cervical cancer cells through the alternative splicing pathway. However, the later RNA binding protein immunoprecipitation (RIP) assay proved that GBP1 was not a direct alternative splicing factor, while the co-immunoprecipitation (CoIP)-mass spectroscopy (MS) assay combined with protein protein interaction (PPI) analysis proved that 8 alternative splicing factors including Heterogeneous Nuclear Ribonucleoprotein K (HNRNPK) were interacting proteins of GBP1. Combined with the existing reports and the results of RNA-seq alternative splicing analysis, it is speculated that GBP1 may regulate the alternative splicing of CD44 protein by binding to interacting protein-HNRNPK, and thus play a role in promoting cancer in cervical cancer.

**Supplementary Information:**

The online version contains supplementary material available at 10.1186/s12967-023-04837-6.

## Introduction

Cervical cancer is the fourth most common cancer in women and remains one of the major public health problems affecting middle-aged women, especially in countries with insufficient resources [1]. Although HPV vaccination has reduced the incidence rate of cervical cancer to some extent. However, the continuous search for new diagnostic or prognostic markers can effectively reduce the mortality of patients [2]. Guanylate binding protein 1 (GBP1) is the most concerned member of the GBP family, originally extracted from interferon (IFN)-induced human fibroblasts, has the unique features of binding with guanosine triphosphate (GTP), guanosine diphosphate (GDP) and guanosine monophosphate (GMP), hence the name [3]. It can mediate host defense, and has the functions of resisting various viruses, bacterias and protozoa pathogens [4–6]. And it also can mediate inflammatory cytokines to inhibit the proliferation, diffusion and migration of endothelial cells, thus playing an anti-angiogenesis role [7]. As a member of the GBP family, it is widely present in mammals and most vertebrates. Studies have found that GBP1 is also widely expressed in various tumors, such as colorectal cancer and prostatic cancer [8, 9]. GBP1 is one of the most potent cellular proteins induced by IFN-γ and can mediate a variety of cellular responses to IFN-γ, including inhibiting proliferation, diffusion, migration and invasion, then exerts anti-tumor activity through it, which may be partly related to defects in angiogenesis of tumors [10, 11]. On the one hand, studies have found that the anti-tumor effect of GBP1 is reflected in colorectal cancer, liver cancer, epithelial ovarian cancer, high-grade serous ovarian cancer and other cancers, and high expression of GBP1 is associated with better survival prognosis in these cancer patients [12–15]. On the other hand, a large number of studies have shown that the high expression of GBP1 in lung cancer could promote the invasion and metastasis of tumor cells, and have certain drug resistance [16, 17]. Ji et al. found that GBP1 was also associated with promoting tumor growth and lymph node metastasis in glioblastoma multiforme and esophageal squamous cell carcinoma, which predicts poor prognosis [18, 19]. These results indicate that GBP1 plays different roles in different kinds of malignant tumors. At present, there are rare reports on the role of GBP1 in cervical cancer. In this study, the bioinformatics methods combined with a large number of in vitro and in vivo experiments were used to fully elucidate the potential biological functions of GBP1 in cervical cancer and conduct in-depth research on its cancer-promoting mechanism. This will lay a foundation for GBP1 as a new therapeutic target for cervical cancer.

## Material and methods

### Expression of GBP1

GBP1 mRNA expression matrices and clinical information data of each tumor tissue and normal tissue were obtained from The Cancer Genome Atlas (TCGA) database (https://tcga-data.nci.nih.gov/tcga/) and Genotype-Tissue Expression (GTEx) database (https://gtexportal.org/), and the two databases were merged. The expression of GBP1 in 33 tumor tissues (TCGA) was compared with that in adjacent tissues (TCGA) and normal tissues (GTEx) by Wilcoxon rank sum test. The expression of GBP1 RNA in 18 types of immune cells and total peripheral blood mononuclear cells (PBMCs), and immunohistochemical verification of GBP1 protein in CESC tissue, were obtained from the HPA database (https://www.proteinatlas.org/). The nTPM represents the standardized level of expression following the internal normalization pipeline and immunohistochemical staining was performed with CBA015450 antibody. Single cell sequencing data (E-MTAB-11948) of cervical cancer samples were obtained from the ArrayExpress database (https://www.ebi.ac.uk/arrayexpress/), the umap R package was used for UMAP analysis, and the cells were annotated using the seurat R package.

### Protein interaction analysis, gene correlation analysis and drug susceptibility analysis of GBP1

Protein–protein interactions (PPIs) network analysis was performed for GBP1 using the STRING database (https://string-db.org/) and GPS-Prot online server (http://gpsprot.org/index.php). In STRING database, the minimum required interaction score set to high confidence (0.700), max number of interactors to show 1 st shell set to no more than 20 interactors, and the rest left the defaults. In GPS-Prot, score was set to 0.6. In the gene correlation analysis, the correlation of GBP1 expression with miRNAs, lncRNAs, mutated genes and protein coding genes was explored. The correlation analysis between GBP1 expression and miRNAs by miRDB (http://www.mirdb.org/), TargetScanHuman (http://www.targetscan.org/vert_80/) and microT-CDS (https://dianalab.e-ce.uth.gr/html/dianauniverse/index.php?r=microT_CDS) databases. Score was set as 1. The correlation of GBP1 expression with lncRNAs and protein coding genes in CESC was performed by obtaining RNAseq data from TCGA database and single gene correlation analysis by stat R package. Subsequently, through the “Target” module of muTarget (https://www.mutarget.com/), select “all somatic mutations” as the somatic mutation type, mutation prevalence at least 3%, and threshold default to explore the top 10 mutated genes affecting GBP1 expression in CESC (n = 286). Finally, the drug sensitivity and gene expression information were downloaded from the Cell Miner database (https://discover.nci.nih.gov/cellminer/analysis.do), and the drug sensitivity analysis of GBP1 was performed using R.

### Correlation between GBP1 expression and tumor microenvironment (TME)

The correlation between GBP1 expression in CESC and immunoinhibitors, immunostimulators, chemokines and chemokine receptors was analyzed by TISIDB database (http://cis.hku.hk/TISIDB/). Download cervical cancer STAR-counts data and clinical information from the TCGA database (https://portal.gdc.com), and ultimately retain samples with RNAseq data and clinical information. To reliably assess immune cell infiltration, we used the immunedeconv R package, which integrates six of the latest algorithms, including TIMER, xCell, MCP-counter, CIBERSORT, EPIC and quanTIseq. Analysis and visualization through the ggClusterNet R package. Among them, immune score, stromal score and tumor microenvironment score were included.

### Enrichment analyses of GBP1-related genes and differentially expressed genes (DEGs)

In order to explore the potential biological functions of GBP1 in CESC, GO and KEGG enrichment analyses were performed on the top 300 related genes based on GBP1 single gene correlation analysis. RNAseq data of GBP1 in single gene difference analysis were obtained from TCGA database and analysis was performed by DESeq2 R package. Gene Set Enrichment Analysis (GSEA) was used to investigate the potential biological function of GBP1-DEGs in CESC, and the normalized enrichment score (NES) > 1.5, false discovery rate (FDR) < 0.25 and p.adjust < 0.05 were considered significantly enriched. GO, KEGG and GSEA were performed by the ClusterProfiler R package.

### Multiplex immunofluorescence (mIF)

On the basis of bioinformatics research, in order to verify the expression of GBP1 in cervical cancer tissues, especially its relationship with T cell infiltration and programmed cell death protein 1 (PD-1)/ Programmed cell death 1 ligand 1 (PD-L1), we purchased tissue chips from Shanghai Outdo Biotech Co., Ltd. Cervical cancer 120-point tissue chip (HUteS120Su01) was placed in the oven at 63 °C and waxed for 1 h. After dewaxing, dilute 10 × repair solution to 1 × working solution, boil at high heat for 3 min, put into slides, continue repairing at low heat for 15–20 min, cool at room temperature, soak in pure water. The slides were placed in a wet box, treated with commercial H_2_O_2_ for 10 min, and cleaned by TBST. Add blocking buffer and incubate for 10 min. The buffer was discarded, diluted polyclonal rabbit α-GBP1 antibody (Proteintech, Chicago, USA, 15303-1-AP) was added, incubated at room temperature for 1 h, and cleaned by TBST. Add secondary antibody, incubate at room temperature for 10 min, and clean TBST. Drop Opal dye diluent (1:100), incubate at room temperature for 10 min, TBST cleaning. The antigen repair steps were repeated, and the pure water was changed to TBST for cleaning to remove the primary and secondary antibodies. Re-dye CD3 (cluster of differentiation 3), PD-1, PD-L1, CK (cytokeratin) indexes, and repeat the above steps. DAPI (4, 6-Diamidino-2-phenylindole) working solution was added, incubated at room temperature for 5 min, cleaned by TBST, the slide was removed, and fluorescent anti-quenching tablet was added to seal the film. GBP1, CD3, PD-1, PD-L1 and CK correspond to fluorescent dyes Opal690, Opal480, Opal520, Opal620 and Opal780, respectively. Vectra Polaris multi-spectral scanning imaging, TissueFAXS Spectra multi-spectral quantitative analysis of panoramic tissue cells, subsequently, Phenochart software and inForm software were used for image processing. Finally, we made full use of the clinicopathological data of 104 cases of cervical cancer and performed the survival analysis in patients with cervical cancer. CK+GBP1+/CK+ (%) was divided into high and low groups by the median method. CK+GBP1+/CK+ (%) represented the percentage of GBP1 positive cells in cervical cancer cells.

### Cell culture

In order to further confirm the effects of GBP1 on cervical cancer through experiments, especially the effects of GBP1 knockdown and overexpression on the proliferation, invasion and apoptosis of cervical cancer cells, we first conducted cell culture assay. Human cervical cancer Caski cell line (Procell, Wuhan, China, CL-0048) was used for GBP1 overexpression assay and GBP1 knockdown assay. Cell culture was performed at 37 °Cand 5% CO_2_ saturated humidity with 10% fetal bovine serum (FBS), 1% 100 U/ml penicillin and 100 U/ml streptomycin in the basal medium. Normal cultured cells were taken, the original culture medium was sucked away, washed with phosphate buffered saline (PBS), digested with pancreatic enzyme for 1–3 min, then digestion was terminated, single cells were blown into with pipette gun, cell suspensions of 15 μl and 20 μl were taken for counting, and the remaining cell suspensions were centrifuged at 1000 rpm for 5 min, then re-suspended with medium, and plates were laid with 4 × 10^5^ cells/well, respectively. 3/6 replicates per group were cultured overnight.

### Small interfering RNA (siRNA) transfection

1.5 μl siRNA with a final concentration of 20 μM was taken, and 100 μl DMEM (Procell, PM150210) was added, and incubated for 3–5 min. Meanwhile, 7.5 μl Liopfectamine RNAiMAX (Invitrogen, Waltham, MA, USA, 13778150) was taken, 100 μl DMEM was added and incubated for 5 min. Subsequently, the diluted siRNA was mixed with Liopfectamine reagent and incubated for 5 min. The above mixture was added into the cell culture medium, and then replaced with fresh medium, 5% CO_2_, and cultured at 37 °C for 48 h after 4–6 h. 48 h after transfection, cells were digested and collected with 0.25% pancreatic enzyme. The siRNA sequence is as follows (5ʹ-3ʹ):

siNC-sense: UUCUCCGAACGUGUCACGUTT

siNC-antisense: ACGUGACACGUUCGGAGAATT

GBP1-Homo-432-sense: CAGUCUCACACUAAAGGAATT

GBP1-Homo-432-antisense: UUCCUUUAGUGUGAGACUGTT

### Lentivirus vector infection

The GBP1 overexpression Lentivirus vector (LV-5-OE-GBP1) was purchased from unibio, Changsha, China. Transcription information: NM_002053. The Caski cells were divided into two groups: OE-NC (empty vector negative control group) and OE-GBP1 (GBP1 overexpressed vector group). According to the multiplicity of cell infection (MOI = 100), the virus was diluted using cell complete culture medium, and the original complete culture medium of the cells was replaced and cultured in 5% CO_2_ at 37 °C. 24 h after infection, the corresponding fresh and complete medium was replaced and placed in 5% CO_2_ in an incubator at 37 °C for 48 h, and the cells were screened by the medium containing purinomycin.

### RNA extraction and quantitative reverse transcription polymerase chain reaction (qRT-PCR) analysis

To verify the success of GBP1 knockdown assay and overexpression assay at GBP1 mRNA level, we performed qRT-PCR analysis. Total RNA was extracted by TRIZOL (Ambion, Texas, USA, 15596-018). The RNA was further purified with two phenol–chloroform treatments, followed by treatment with RQ1 DNase (Promega, Madison, WI, USA) to remove the DNA. The absorbance of 260 nm/280 nm (A260/A280) was measured by NanoPhotometer N50 (IMPLEN, NanoPhotometer N50), and the quality and quantity of purified RNA were re-determined. The integrity of RNA was further verified by 1.0% agarose gel electrophoresis. GAPDH was used as a control gene to evaluate the effects of GBP1 overexpression and GBP1 knockdown. cDNA synthesis was performed according to standard procedures, and qRT-qPCR was performed on ABI QuantStudio5 using Hieff™ qPCR SYBR®Green Master Mix (Low Rox Plus; YEASEN, China, 11202ES08). The concentration of each transcript was then normalized to GAPDH (glyceraldehyde-3-phosphate dehydrogenase) mRNA levels using the 2^−ΔΔCT^ method [20]. Primer information is as follows (5ʹ to 3ʹ):

GBP1-F CTCCAGACAGACCAGACT

GBP1-R CGTTCTCCATCTTCTCAGT

Hsa GAPDH_F GGTCGGAGTCAACGGATTTG

hsa GAPDH_R GGAAGATGGTGATGGGATTTC

### Western blot analysis

To verify the success of GBP1 knockdown assay and overexpression assay at GBP1 protein level, we performed western blot analysis. Caski cells were lysed in an Ice-cold Wash Buffer (1 × PBS, 0.1% SDS, 0.5% NP-40 and 0.5% sodium deoxycholate) with a protease inhibitor mixture (Roche) and incubated on ice for 30 min. Then, add the 1 × SDS sample buffer and boil in boiling water for 10 min. Subsequently, the extracted proteins were separated by 10% sodium dodecyl sulfate-polyacrylamide gel electrophoresis (SDS-PAGE). Then, the proteins were blocked in TBST buffer containing 5% skimmed milk powder (20 mM tris buffered brine and 0.1% Tween-20) for 1 h. The sample was 30 μg per well, and the total protein volume was 100 μl. Then, the proteins were stayed overnight with anti-Flag antibody (Sigma, China, F1804, 1:1000), anti-GBP1 antibody (ABclonal, China, 1:1000, A3879) and GAPDH (glyceraldehyde-3-phosphate dehydrogenase) (Proteintech, China, 60004-1-IG) at 4 °C. GAPDH (glyceraldehyde-3-phosphate dehydrogenase) (Proteintech, China, 60004-1-IG) was used as an internal parameter, and Flag (ABclonal, China, AE005) was used as a label protein. Then, the proteins were incubated with horseradish peroxidase (HRP)-conjugated goat anti-mouse IgG or anti-rabbit IgG (Proteintech, China, SA00001-1/2, 1:500) at room temperature for 1 h. Finally, proteins were incubated with an enhanced chemiluminescence (ECL) reagent (Bio-Rad, 170506) to detect the binding secondary antibody.

### CCK8 assay, transwell invasion assay and cell apoptosis assay

Based on the above experiments, further, CCK8 assay, transwell invasion assay and cell apoptosis assay were used to explore the effects of GBP1 knockdown and overexpression on proliferation, invasion and apoptosis of cervical cancer cells. The proliferation capacity of Caski cells was evaluated using Cell Counting Kit-8 (CCK8) assay. Normal cultured cells were taken, and after digestion, 10^4^ cells/holes (96 holes) were used to lay plates, 3 multiple holes, and 3/6 replicates per group. 10 µl CCK8 solution (MCE, USA, HY-K0301) was added to each well and cultured in 5% CO2 at 37 °C for 3 h. Absorbance was measured at 450 nm using an enzyme-linked immunosorbent assay plate reader (FC, Thermo). In vitro invasion tests were conducted in the transwell chamber (3422, Corning, USA). A transwell chamber with an 8 µm filter was pre-coated with a thin layer of Matrigel (356234, BD Biosciences, USA), diluted with serum-free medium at 1:8, and incubated with 100 µm diluted Matrigel at 37 °C and 5% CO_2_ for 1 h. Remove unsolidified supernatant. 0.2 ml serum-free medium 5 × 10^5^ cells were added to the insert, and transwell cavity containing 600 µl 10% FBS (10091148, Gibco, China) was inserted into the lower cavity as a chemical inducer, and incubated at 37 °C and 5% CO_2_ for 48 h. The remaining cells on the surface of the upper membrane of the insert were then removed with a cotton swab, and the total number of cells invading the lower cavity was fixed with 4% paraformaldehyde (P0099, Beyotime, China) for 20 min, and then stained with 0.1% crystal purple (C0121, Beyotime, China). The infiltrated cells were observed and counted under inverted microscope (MF52-N, Mshot, China) at 200 magnification. Apoptosis was detected by flow cytometry (Beckman). 10^5^ Caski cells were inoculated in 24-well culture plates and cultured at 37 ℃ and 5% CO2 for 24 h. Double staining was used for Fluor647-conjugated Annexin V and PI (4A Biotech Co. Ltd, Beijing, China).

### Animal model

The experiment selected female nude mice aged 6–8 weeks and fed them for one week to start the animal experiment. The experiment was divided into Caski-OE-NC group and Caski-OE-GBP1 group, with 5 animals in each group. After the selection of Caski cells for two times, 4 × 10^6^ cells were injected subcutaneously under the armpit, and the state of nude mice was observed for 17 days. The tumor volume (mm^3^) was measured with the formula

$$ {\text{V}} = 0.52 \times {\text{L}} \times {\text{W}}^{2} $$

L is the longest diameter of the tumor, W is the shortest diameter of the tumor.

### RNA sequencing and alternative splicing analysis

In order to explore the cancer promoting mechanism of GBP1 in cervical cancer, we conducted RNA sequencing to find the alternative splicing pathway. Total RNA was treated with RQ1 DNase (Promega) to remove DNA. The quality and quantity of purified RNA was determined by measuring 260/280 nm (A260/A280) absorbance using smartspec plus (BioRad). RNA integrity was further verified by 1.5% agarose gel electrophoresis. RNA-seq libraries were prepared using the KAPA strand mRNA-Seq Kit For Illumina®Platforms (KAPA,Washington, USA, KK8544) with 1 μg total RNA from each sample. Polyadenylated mRNA was purified and segmented by VAHTS mRNA capture beads (N401-01). The fragment mRNA is converted to double-stranded cDNA. After terminal repair and A-caudation, the DNA is attached to the Diluted Roche Adaptor (KK8726). The junction products were purified, scaled to 300–500 bps, amplified and purified, quantified, stored at − 80 °C, before sequencing. The strand labeled with dUTP (the second cDNA strand) was not amplified, allowing specific sequencing. For high-throughput sequencing, the library was applied to the Illumina Novaseq 6000 system for 150 nt peer sequencing. Each sample in the RNA-seq data was aligned to unique mapped reads on the reference genome for alternative splicing analysis. The splice junction of each sample was analyzed with TopHat2 [21]. At the same time, GO and KEGG analysis were performed for the involved alternative splicing genes.

### Improved RNA immunoprecipitation sequencing (iRIP-seq)

To further explore whether GBP1 is a alternative splicing factor, we performed iRIP-seq. Caski cells were irradiated once with 400 mJ/cm^2^ and lyzed in ice-cold wash buffer (1 × PBS, 0.5% SDS, 0.5% NP-40 and 0.5% deoxychocolate-sodium). Add 400 U/mL RNase inhibitor (Takara) and protease inhibitor mixture (Bimake) and incubate on ice for 30 min. RQI (Promega, 1 U/μl) was added until the final concentration was 0.1 U/μl, and incubated in 37 °C hot block for 30 min. The mixture was then strongly vibrated and centrifuged at 4 °C at 13,000×*g* for 15 min to remove cell debris. The RNA is then cut with the enzyme mase. Add EDTA to stop digestion. The supernatant was incubated with 10 μg Flag antibody (Sigma, China, F7425) and control IgG antibody (AC005) at 4 °C overnight. Immunoprecipitation was further incubated with protein A/G Dynabeads (Thermo Scientific) at 4 °C for 2 h. After applying the microsphere to the magnet and removing the supernatant, Pyrolysis buffer (1 × PBS, 0.1% SDS, 0.5% NP-40 and 0.5% sodium deoxycholate), high-salt buffer (250 mM Tris 7.4, 750 mM NaCl, 10 mM EDTA, 0.1% SDS, 0.5%) were used, respectively NP-40 and 0.5 sodium deoxycholate) and PNK buffers (50 mM Tris, 20 mM EGTA and 0.5% NP-40) were washed twice. The beads were re-suspended with elution buffers (50 mM Tris 8.0, 10 mM EDTA and 1% SDS). Incubated in a hot block at 70 °C for 30 min, immunoprecipitated RNA and vortex RBP are released. Remove the magnetic beads on the separator and transfer the supernatant into a clean 1.5 ml microtubule. Proteinase K (Sangon Biotech) was added with 10% input (without immunoprecipitation), immunoprecipitation RNA cross-linked RBP, and the final concentration was 1.2 mg/ml. Incubate at 55 °C for 120 min. Use phenol: chloroform: isoamyl alcohol (25: 24: 1, pH < 5) Reagent (Solarbio) purified RNA. The cDNA library was prepared using the KAPA RNA Hyper Prep Kit (KAPA) according to the manufacturer's procedures. For high-throughput sequencing, the libraries were prepared according to manufacturer's instructions and applied to the Illumina NovaSeq6000 system for 150 nt paired end sequencing. After we aligned reads with the genome, only uniquely mapped reads were applied to the following analysis. The “ABLIRC” strategy was used to identify TTP binding regions on the genome [22]. Overlapping reads of at least 1 bp were clustered as peaks. For each gene, we use computational simulations to randomly generate the same number and length of reads as the peak reads. The output reads were further mapped to the same genes to generate random maximum peak heights from overlapping reads. The whole process was repeated 500 times. All observed peak heights are higher than random maximum peaks (p-value < 0.05). The “Piranha” strategy is to select a fixed length (xx nt) as a unit (bin) according to sequencing depth and coverage, and calculate the number of reads per bin. The reads distribution in the simulated data was used as background noise, and the position where the reads distribution was significantly higher than the background was found based on zero truncated negative binomial (ZTNB). In this process, each bin will get a p-value, according to the p-value < 0.05), screening significance, the true binding peak was obtained [23]. The GBP1 and Input samples were simulated and analyzed respectively, and the GBP1 peaks overlapping with the Input peaks were removed.

### Co-immunoprecipitation (CoIP)-mass spectroscopy (MS)

Based on the results of iRIP-seq, in order to find the alternative splicing factors binding to GBP1, and then play a role in promoting cancer through the alternative splicing pathway, we conducted a CoIP and MS combined assay. The CoIP-MS experiment was conducted with the assistance of Beauty of Life Technology Co., LTD. (Wuhan, China). The cells were cleaved on ice for 1 h with NP-40 buffers (50 mM Tris (pH 7.4), 150 mm NaCl, and 1% NP-40), and fresh protease and phosphatase inhibitors were added. After the lysate was centrifuged at 1000×*g* at 4 °C for 10 min, the supernatant was collected. Incubate at 4 °C for 2 h with 10 μg anti-Flag antibody (Sigma, China, F1804, 1:1000) or 5 μg Mouse IgG (Millipore, China, 12-371, 1:1000). Then 20 μl protein A/G magnetic beads were added and incubated at 4 °C for another 1 h. After washing three times with washing solution (50 mM Tris (pH 7.4), 150 mM NaCl, 1 mM EDTA and newly added protease inhibitor), elution was performed for western blotting. The primary antibody was anti-Flag antibody and mouse IgG, the secondary antibody was anti-mouse or anti-rabbit (Proteintech, China, SA00001-1/2, 1:500), and GAPDH (ATA, China, ATPA00013Rb, 1:500) was used as the internal reference. After denaturation, the protein was isolated by SDS-PAGE and transferred to the PVDF membrane (Millipore, Massachusetts, USA, ISEQ00010), sealed with 5% skim milk at room temperature for 1 h, diluted with mono (5% milk dilution), and incubated at 4 °C overnight. The film was washed and incubated with TBST diluent at room temperature for 45 min. Clean the membrane with TBST. The ECL luminous solution was prepared at 1: 1 and exposed with an exposure meter. At the same time, protein silver staining was performed. After the SDS-PAGE is completed, the gel is fixed on a shaker for 15 min at room temperature and then transferred to the sensitizer. Add the dyeing solution and stir on a shaker for 30 min. After staining, it is rinsed and incubated in the developer until the bands are clearly visible and imaged. After the success of CoIP was confirmed by immunoblotting and silver staining experiments, different bands were cut and sent to Novogene Bioinformation Technology Inc. Mass spectrometry was performed. Then the original data after quality control were analyzed by bioinformatics.

### Statistical analysis

GraphPad Prism v.9.0 software and R v4.1.3 software were used for analysis. Comparisons between the two groups were made using the unpaired two-tailed Student’s t test or χ^2^ test. When the data is not normally distributed, the Wilcoxon rank-sum test is used. survival analysis was performed using log-rank test, and Kaplan–Meier survival curve was drawn using survminer and survival R software packages. Spearman or Pearson correlation analysis was used for correlation analyses. The results were expressed as the mean ± SEM of at least three replicates. Each p value < 0.05 was considered statistically significant.

## Results

### The expression profile of GBP1

**Fig. 1:**
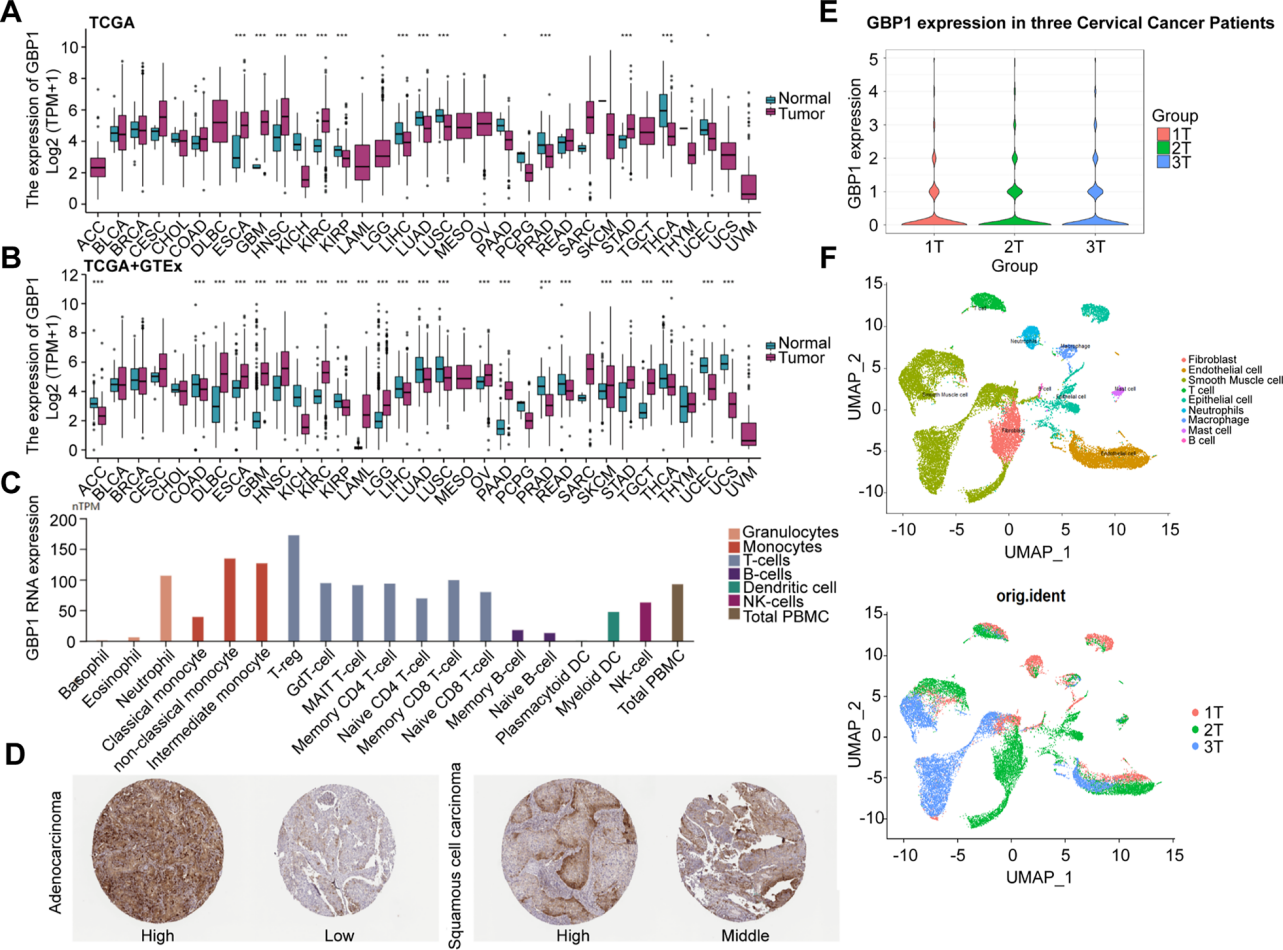
GBP1 expression profile. **A** Differential GBP1 expression levels in 33 tumors and adjacent normal tissues in TCGA database, *p < 0.05, **p < 0.01, ***p < 0.001. **B** Differential GBP1 expression levels in 33 tumors and normal tissues in TCGA database and GTEx database, *p < 0.05, **p < 0.01, ***p < 0.001. **C** GBP1 RNA normalized expression (nTPM) levels for 18 types of immune cells and total peripheral blood mononuclear cells (PBMCs) in the HPA database. **D** Immunohistochemical staining of cervical squamous cell carcinoma and endocervical adenocarcinoma (CESC) tissue was performed with CBA015450 antibody from the HPA database for GBP1. **E** Violin plot of GBP1 expression in three CC patients. **F** Uniform Manifold Approximation and Projection (UMAP) plot from three CC patients. *CC* Cervical cancer

TCGA data analysis showed that GBP1 was widely expressed in Cervical squamous cell carcinoma and endocervical adenocarcinoma (CESC) tissue, but did not have significant differences compared with normal tissue. The expression level of GBP1 in esophageal carcinoma (ESCA), glioblastoma multiforme (GBM), head and neck squamous cell carcinoma (HNSC), kidney renal clear cell carcinoma (KIRC) and stomach adenocarcinoma (STAD) tissues was higher than that in normal tissues (p < 0.001), in kidney chromophobe (KICH), kidney renal papillary cell carcinoma (KIRP), liver hepatocellular carcinoma (LIHC), LUAD, lung squamous cell carcinoma (LUSC), PRAD, thyroid carcinoma (THCA), pancreatic adenocarcinoma (PAAD) (p < 0.05) and uterine corpus endometrial carcinoma (UCEC) (p < 0.05) tissues was lower than in normal tissues (p < 0.001) (Fig. 1A). Because TCGA database has lesser normal tissue data (n = 727), we combined the normal tissue data (n = 5242) from GTEx database, compared with the tumor data (n = 9807) of TCGA database. The results showed that the expression levels of GBP1 were higher in lymphoid neoplasm diffuse large B-cell lymphoma (DLBC), ESCA, GBM, HNSC, KIRC, acute myeloid leukemia (LAML), low-grade glioma (LGG), ovarian serous cystadenocarcinoma (OV), PAAD, skin cutaneous melanoma (SKCM), STAD and testicular germ cell tumors (TGCT) tissues than normal tissues, and lower in adrenocortical carcinoma (ACC), colon adenocarcinoma (COAD), KICH, KIRP, LIHC, LUAD, LUSC, PRAD, rectum adenocarcinoma (READ), THCA, UCEC and uterine carcinosarcoma (UCS) tissues (Fig. 1B). We also explored GBP1 RNA expression in 18 immune cell types and total peripheral blood mononuclear cells (PBMCs). The results showed that GBP1 RNA was highest in regulatory T cells (T-regs) and monocytes, and was expressed in most immune cells (Fig. 1C). In addition, immunohistochemical results of HPA database showed that GBP1 protein could be detected in both cervical adenocarcinoma and cervical squamous cell carcinoma, presenting low to high staining results. GBP1 was commonly expressed in CESC tissue (Fig. 1D). Using the single-cell sequencing data of cervical cancer samples (E-MTAB-11948), UAMP dimensional reduction analysis was performed on 3 patients with cervical cancer expressing GBP1, and the results showed that GBP1 was expressed in cells such as fibroblasts, T cells and neutrophils (Fig. 1E, F).

### Protein interaction analysis, gene correlation analysis and drug susceptibility analysis of GBP1 expression

**Fig. 2:**
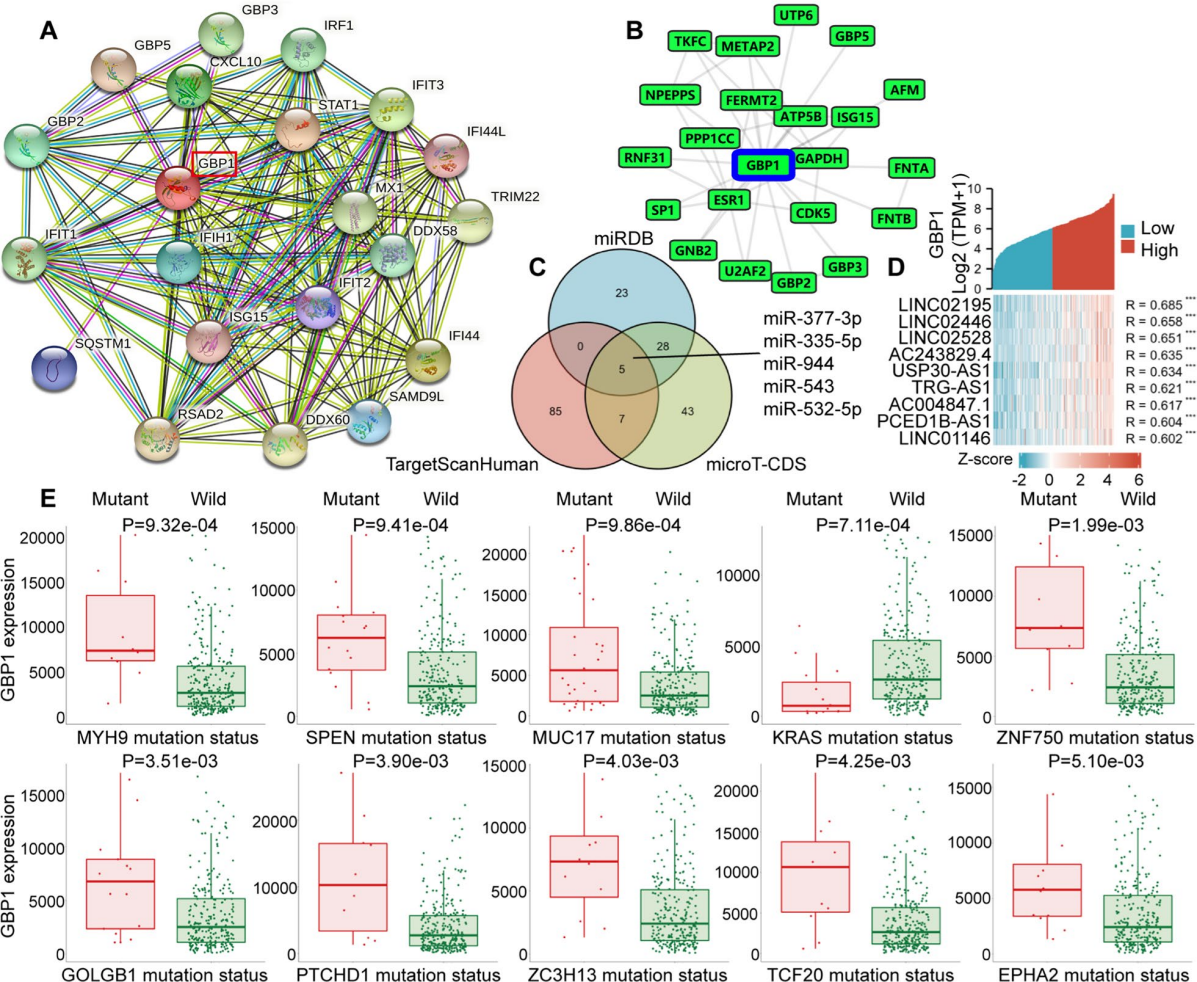
**A** GBP1-interacting proteins network analysis in the STRING database. **B** GBP1-interacting proteins network analysis in the GPS-Prot online server. **C** Venn diagram showed miRNAs associated with GBP1 genes from miRDB, TargetScanHuman and microT-CDS databases. **D** Co-expression heat map showed lncRNAs associated with GBP1 genes from TCGA database. **E** The top 10 mutated genes that affect GBP1 expression upregulation or downregulation in the muTarget database

**Fig. 3:**
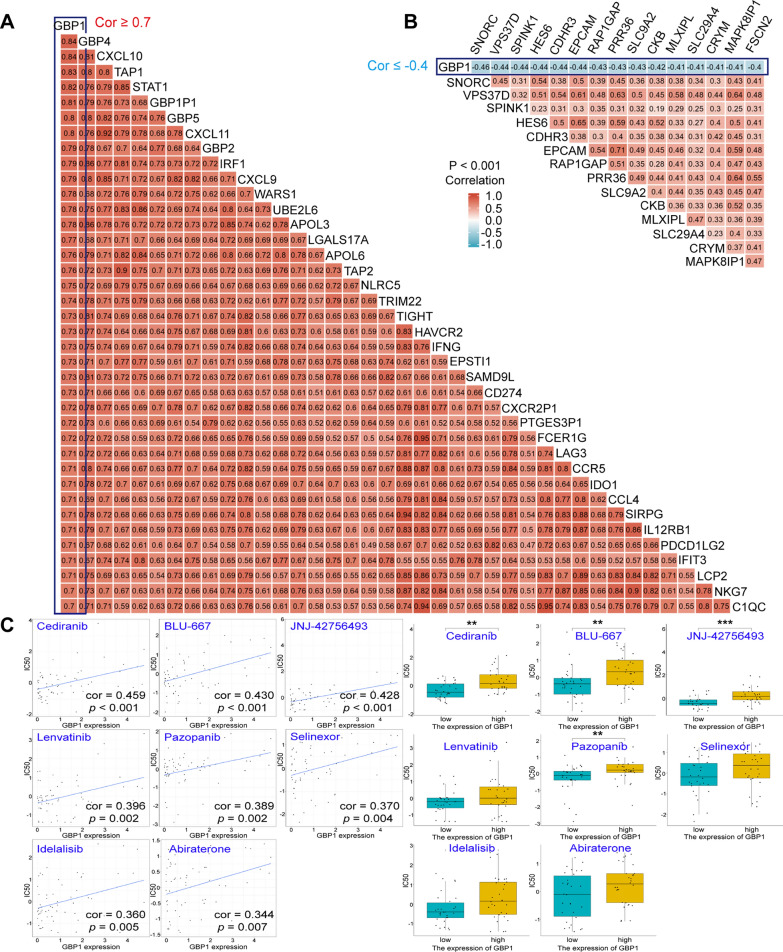
**A** Heat map of genes positively associated with GBP1. The portion with correlation coefficients ≥ 0.7 was shown. **B** Heat map of genes negatively associated with GBP1. The portion with correlation coefficients ≤ − 0.4 was shown. p < 0.001. **C** Drug sensitivity analysis of GBP1. IC50, 50% inhibitory concentration. **p < 0.01, ***p < 0.001

To explore the proteins that interact with GBP1, we used two PPI databases. In STRING database, average local clustering coefficient was 0.877, PPI enrichment p-value < 1.0e−16. The results showed that GBP1 interacted with 21 proteins including STAT1, IFI44, IFIT3, CXCL10 and GBP2 (Fig. 2A). In GPS-Prot database, min conf score was 0.6, and the result showed that GBP1 interacted with 20 proteins including GAPDH, NPEPPS and U2AF2 (Fig. 2B). Gene correlation analysis included the correlation of GBP1 with miRNAs, lncRNAs, mutated genes and protein-coding genes. The miRNAs involved in GBP1 regulation were explored in miRDB, TargetScanHuman and microT-CDS databases. The results showed that GBP1 was the target gene of miR-377-3p, miR-335-5p, miR-944, miR-543 and miR-532-5p (Fig. 2C). We also used CESC RNAseq data in TCGA database to explore lncRNAs related to GBP1 expression. The single gene co-expression heat map showed lncRNAs with correlation coefficients above 0.6, including 9 lncRNAs such as LINC02195, LINC02446 and LINC02528 (Fig. 2D). In addition, according to the muTarget database result, in CESC (n = 286), mutated genes affecting GBP1 expression including the top 10 genes such as MYH9, SPEN, MUC17, KRAS and ZNF750. In other words, GBP1 expression in the mutated group of these genes was significantly different from that in the wild group (Fig. 2E). Finally, we also used TCGA database data to deeply explore the correlation between GBP1 and genes such as protein-coding genes in CESC. The positive correlation part showed the genes with Cor ≥ 0.7, and the negative correlation part showed the genes with Cor ≤ − 0.4. The results showed that GBP1 was significantly positively correlated with genes such as GBP4, CXCL10, TAP1, STAT1 and GBP1P1 (Fig. 3A), and significantly negatively correlated with genes such as SNORC, VPS37D, SPINK1, HES6 and CDHR3 (Fig. 3B). Drug sensitivity analysis showed that the expression of GBP1 was positively correlated with the IC50 (half maximal inhibitory concentration) of eight drugs, including Cediranib, BLU-667 and JNJ-42756493, that is, as the expression of GBP1 increased, the stronger the resistance of cervical cancer cells to drugs. However, in the GBP1 high expression group and GBP1 low expression group, only four drugs, Cediranib, BLU-667, JNJ-42756493 and Pazopanib, showed significant differences in IC50 (Fig. 3C).

### Correlation between GBP1 expression and tumor microenvironment

**Fig. 4:**
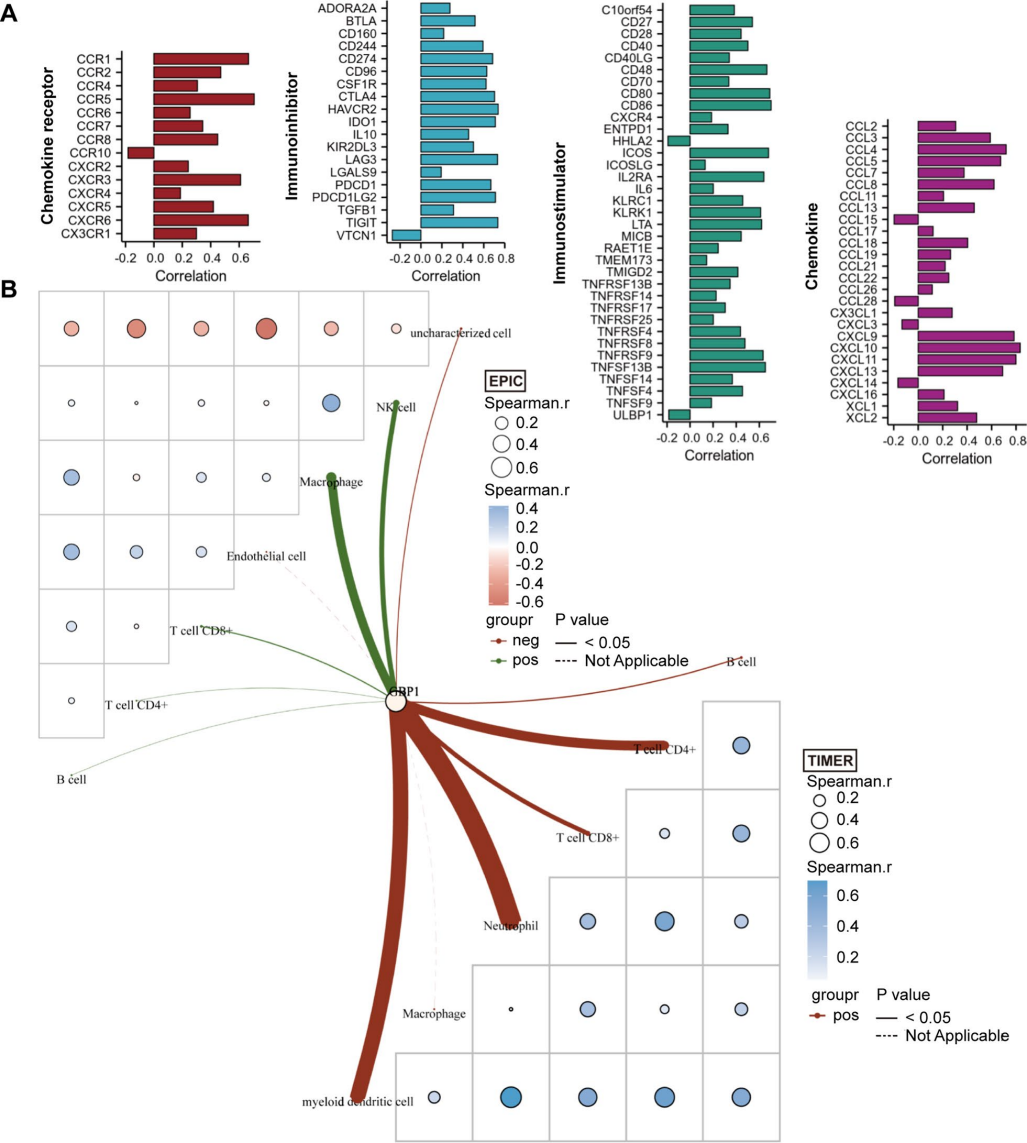
Correlation of GBP1 expression with tumor microenvironment in CESC. **A** Correlation of GBP1 expression with immunoinhibitors, immunostimulators, chemokines and chemokine receptors. p < 0.05. **B** Correlation between GBP1 expression and infiltrating immune cells in EPIC and TIMER algorithms. The heat map represents the correlation analysis of immune score itself, the lines represent the correlation between GBP1 expression and immune score, and the thicker the lines are, the stronger the correlation is

**Fig. 5:**
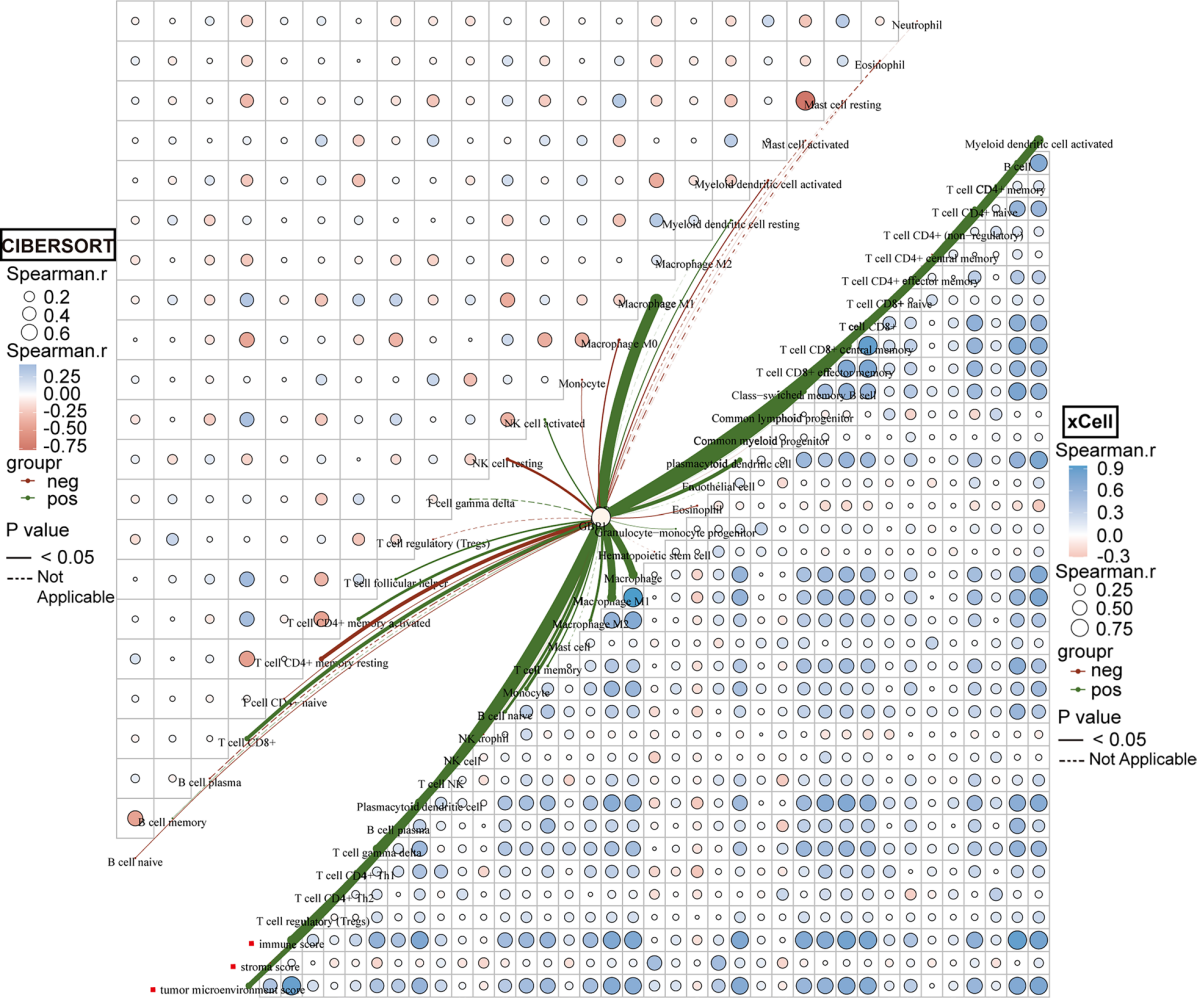
Correlation between GBP1 expression and infiltrating immune cells in CIBERSORT and xCell algorithms. The green line represents a positive correlation between GBP1 expression and immune scores, and the thicker the lines are, the stronger the correlation is

We first used TISIDB database to analyze the correlation between GBP1 expression and two immunomodulators, chemokines and their receptors. The bar chart only showed the statistically significant part of the correlation, most of which showed positive correlation (p < 0.05). The results showed that GBP1 expression was significantly correlated with 19 immunoinhibitors including ADORA2A, and the correlation coefficients with CD274, CD96, CSF1R, CTLA4, HAVCR2, IDO1, LAG3, PDCD1, PDCD1LG2 and TIGIT were above 0.6. GBP1 expression was significantly correlated with 35 immunostimulators including C10orf54, and the correlation coefficients with CD48, CD80, CD86, ICOS, IL2RA, KLRK1, LTA, TNFRSF9 and TNFSF13B were above 0.6. GBP1 expression was significantly correlated with 26 chemokines including CCL2, and the correlation coefficients with CCL4, CCL5, CCL8, CXCL9, CXCL10, CXCL11 and CXCL13 were above 0.6. Then, GBP1 expression was significantly correlated with 14 chemokine receptors including CCR1, and the correlation coefficients with CCR1, CCR5, CXCR3 and CXCR6 were above 0.6. (Fig. 4A). Subsequently, in order to reliably evaluate the infiltration of immune cells, we used the immunedeconv R package on the data from TCGA, which includes six algorithms, namely TIMER, xCell, MCP-counter, CIBERSORT, EPIC and quanTIseq. The heat map in the butterfly diagram showed the correlation of each infiltrating immune cell itself. Blue is positive correlation, red is negative correlation, and the larger the circle, the stronger the correlation. The lines showed the correlation between GBP1 expression and invasive immune cells, the thicker the lines indicated the stronger the correlation, and the solid lines showed statistical significance. The results showed that GBP1 expression was the most correlated with macrophages in the EPIC algorithm, while the expression of GBP1 was the most correlated with neutrophils, Myeloid dendritic cells (MDCs) and CD4+ T cells in the TIMER algorithm (Fig. 4B). In CIBERSORT algorithm, the results showed that the expression of GBP1 was the most correlated with macrophage M1. In the xCell algorithm, the results showed that the expression of GBP1 was the most correlated with activated MDCs, central memory CD8+ T cells (Tcm CD8), macrophage and gamma-delta T cells (γδ T). Moreover, we found that GBP1 had a strong positive correlation with immune score and tumor microenvironment score (Fig. 5). In the MCP-counter algorithm, the results showed that GBP1 expression was the most correlated with natural killer (NK) cells, T cells, monocytes and macrophages, and was positively correlated with cytotoxicity score. In the quanTIseq algorithm, the results showed that GBP1 expression had the strongest correlation with CD8+ T cells (Additional file 1: Figure S1).

### Enrichment analyses of GBP1-related genes and differentially expressed genes (DEGs)

**Fig. 6:**
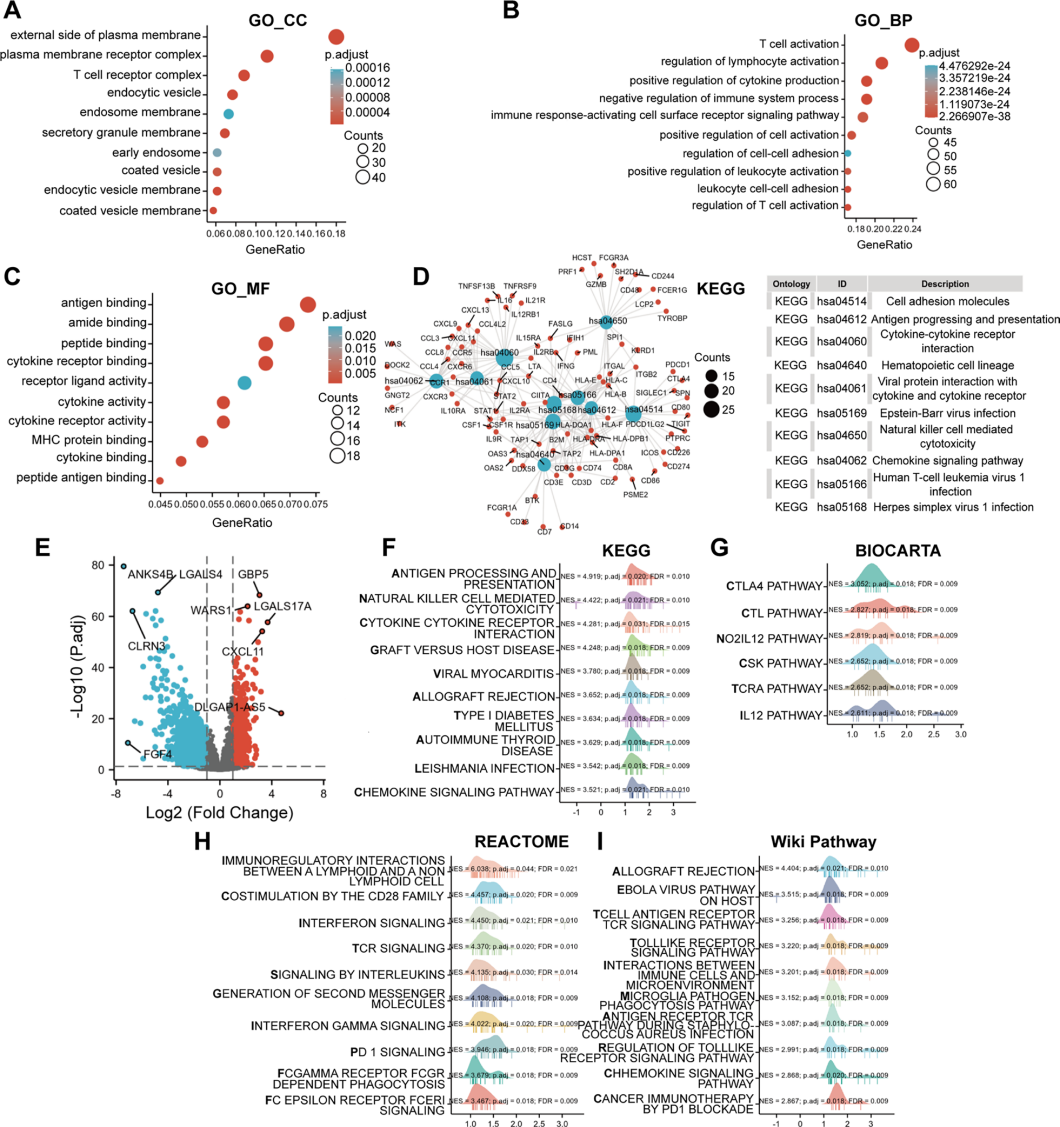
Enrichment analysis for the top 300 related genes and DEGs of GBP1 in CESC. **A** Top 10 terms in CCs from GO enrichment analysis of GBP1 related genes. **B** Top 10 terms in MFs from GO enrichment analysis of GBP1 related genes. **C** Top 10 terms in BPs from GO enrichment analysis of GBP1 related genes. **D** KEGG enrichment analysis of GBP1 related genes showed the top 10 KEGG pathways. **E** Volcanic map of GBP1-DEGs. **F** Top 10 KEGG pathways from GSEA of GBP1-DEGs. **G** Biocarta pathways from GSEA of GBP1-DEGs. **H** Top 10 reactome pathways from GSEA of GBP1-DEGs. **I** Top 10 wiki pathways from GSEA of GBP1-DEGs. *DEGs* differentially expressed genes, *CC* cellular component, *MF* molecular function, *BP* biological process, *GSEA* Gene set enrichment analysis

Firstly, GBP1 single gene correlation analysis and single gene difference analysis were performed on the data from TCGA database, and GO, KEGG enrichment analyses and GSEA were performed respectively. GO enrichment of the top 300 related genes showed that they were significantly enriched in cellular components (CCs) such as external side of plasma membrane, plasma membrane receptor complex, T cell receptor complex, endocytic vesicle and endosome membrane (Fig. 6A), in molecular functions (MFs) such as antigen/amide/cytokine receptor/peptide binding and receptor ligand activity (Fig. 6B), and in biological processes (BPs) such as T cell activation, regulation of lymphocyte activation, negative regulation of immune system process, positive regulation of cytokine production and immune response-activating cell surface receptor signaling pathway (Fig. 6C). KEGG enrichment of related genes showed that they were significantly enriched in pathways such as cytokine–cytokine receptor interaction, cell adhesion molecules, herpes simplex virus 1 (HSV-1)/Epstein–barr virus (EBV) infection and chemokine signaling pathway, and the genes enriched in the top 10 pathways were shown (Fig. 6D). The volcano map of GBP1-differentially expressed genes was shown in Fig. 6E, among which the differences of GBP1 with ANKS4B, CLRN3, GBP5, LGALS17A and CXCL11 genes were the most significant. GSEA results showed that GBP1-DEGs were significantly enriched in KEGG pathways such as antigen processing and presentation, NK cell mediated cytotoxicity, cytokine–cytokine receptor interaction, graft versus host disease (GVHD) and viral myocarditis (Fig. 6F), in Biocarta pathways such as CTLA4, cytotoxic lymphocyte (CTL), NO2-IL12, C-src tyrosine kinase (CSK), TCRA and IL12 (Fig. 6G), in Reactome pathways such as immunoregulatory interactions between a lymphoid and a non lymphoid cell, costimulation by the CD28 family, interferon (IFN)/T cell antigen receptor (TCR)/interleukin (IL) signaling (Fig. 6H), in Wiki pathways such as allograft rejection, Ebola virus pathway on host, TCR/Toll-like receptor (TLR) signaling and interactions between immune cells and microRNAs in tumor microenvironment (Fig. 6I).

### GBP1 expression was positively correlated with PD-L1 expression in cervical cancer

**Fig. 7:**
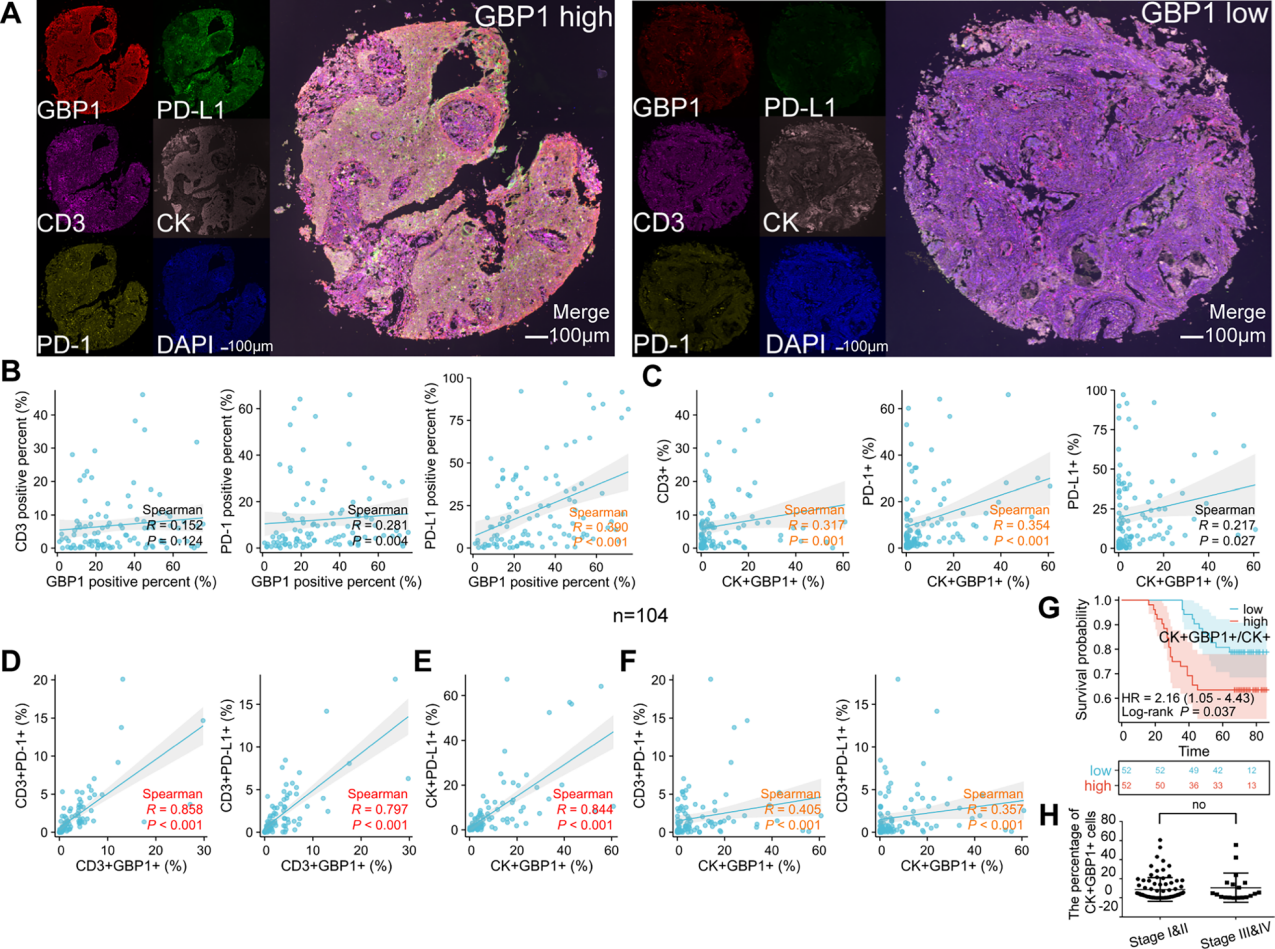
Expression of GBP1, CD3, PD-1, PD-L1 and CK in 104 cervical cancer were detected using multiplexed immunofluorescence. **A** Representative costaining images of GBP1, CD3, PD-1, PD-L1 and CK in the GBP1 high and low expression. Scale bars: 100 µm. **B** Correlation between the GBP1 positive (GBP1+) percent (%) and CD3/PD-1/PD-L1 positive percent (%) in 104 CC. **C** Correlation between the CK+GBP1+ (%) and CD3+/PD-1+/PD-L1+ (%) in 104 CC. **D** Correlation between the CD3+GBP1+ (%) and CD3+PD-1+/CD3+PD-L1+ (%) in 104 CC. **E** Correlation between the CK+GBP1+ (%) and CK+PD-L1+ (%) in 104 CC. **F** Correlation between the CK+GBP1+ (%) and CD3+PD-1+/CD3+PD-L1+ (%) in 104 CC. **G** Survival analysis showing the relationship between CK+GBP1+/CK+ levels and the OS of patients in 104 CC patients. **H** The percentage of CK+GBP1+ cells in stage I&II and stage III&IV CC tissues were compared. CK+GBP1+ represents CC cells expressing GBP1. CD3+GBP1+ represents tumor infiltrating lymphocytes expressing GBP1. CK+GBP1+/CK+ (proportion of CK+GBP1+ in CK+) represents the proportion of GBP1 expressed CC cells in CC cells. *CC* cervical cancer, *OS* overall survival

**Fig. 8:**
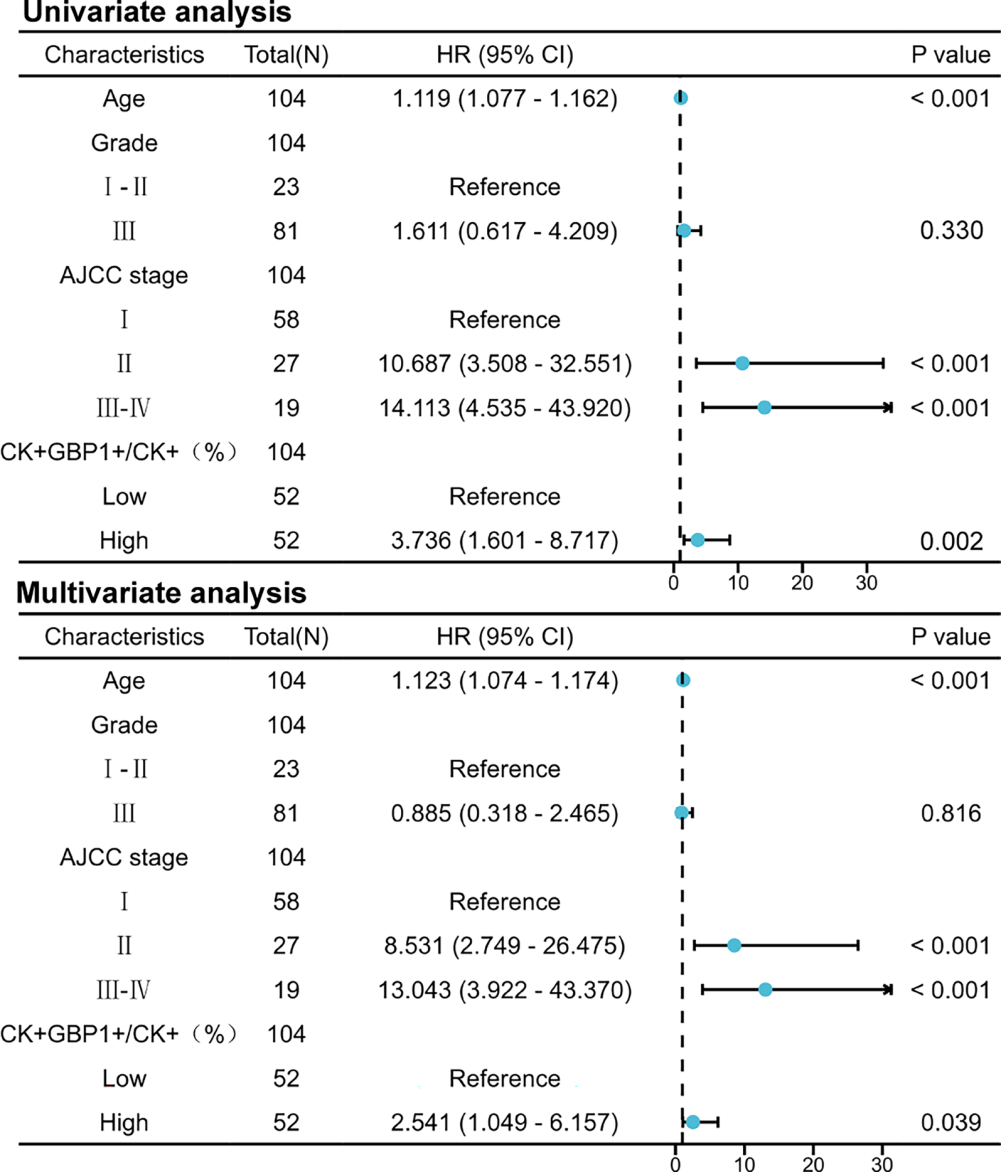
Univariate Cox analysis and multivariate Cox analysis of prognosis of patients with cervical cancer were shown in forest map

To explore the correlation between GBP1 expression and PD-1 and PD-L1 expression in cervical cancer. We detected the expression of GBP1, CD3, PD-1, PD-L1 and CK by polychromatic immunofluorescence staining in 104 cases of cervical cancer. Since most tumor infiltrating lymphocytes (TILs) express CD3, we default CD3+ cells in cervical cancer tissue to TILs and CK+ cells to cervical cancer cells. We found that GBP1+ cell percentage was positively correlated with PD-L1+ cell percentage (Fig. 7A, B). Further analysis showed that the percentage of CK+GBP1+ cells was weakly correlated with the percentage of CD3+ or PD-1+ cells (Fig. 7C). However, the percentage of CD3+GBP1+ cells was strongly positively correlated with the percentage of CD3+PD-1+ or CD3+PD-L1+ cells, indicating that the expression of GBP1 on TILs was strongly correlated with the expression of PD-1 and PD-L1 (Fig. 7D). The percentage of CK+GBP1+ cells was strongly positively correlated with the percentage of CK+PD-L1+ cells, indicating that the expression of GBP1 on the surface of cervical cancer cells was strongly correlated with the expression of PD-L1 (Fig. 7E). In addition, we cross-analyzed the correlation between GBP1 on cervical cancer cells and PD-1 and PD-L1 on TILs, and the results showed that there was also a certain correlation (Fig. 7F). Finally, we conducted data processing for the index CK+GBP1+/CK+ (%) and carried out survival analysis through the median method. Result showed that a higher proportion of GBP1+ cervical cancer cells to cervical cancer cells was associated with poorer OS. In other words, this showed that the number of GBP1-positive cervical cancer cells in cervical cancer cells had a certain impact on the survival of cervical cancer patients. (Fig. 7G). However, there was no statistical difference in the percentage of CK+GBP1+ cells between early cervical cancer and advanced cervical cancer tissues (Fig. 7H). In order to verify whether CK+GBP1+/CK+ (%) is an independent factor affecting the prognosis of patients with cervical cancer, univariate and multivariate Cox analyses were subsequently performed. The result showed that CK+GBP1+/CK+ (%) was an independent prognostic factor (Fig. 8).

### GBP1 Knock-down expression can inhibit the proliferation and invasion of cervical cancer cells and promote cell apoptosis

**Fig. 9:**
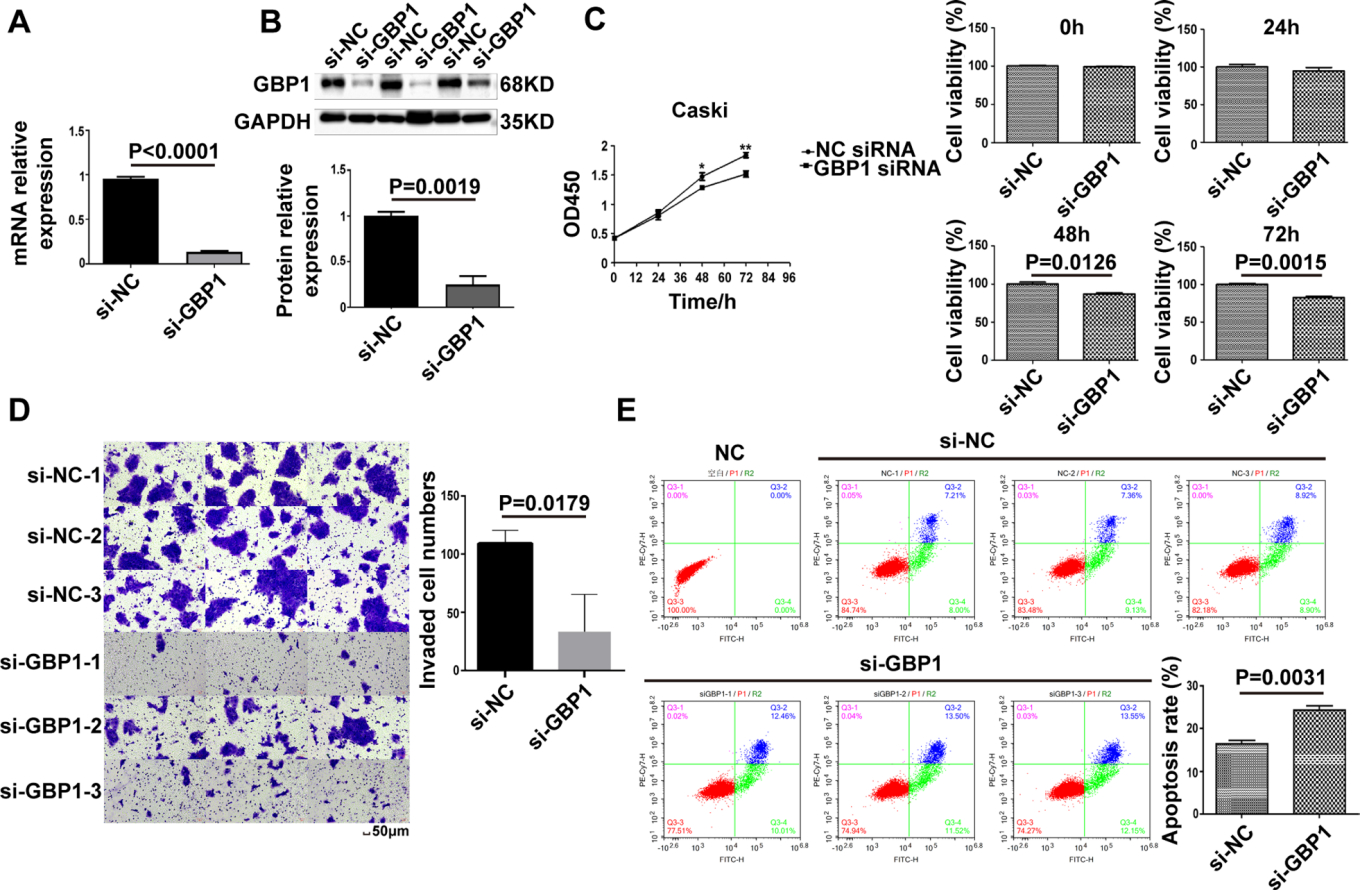
GBP1 silencing can inhibit the proliferation and invasion of cervical cancer cells and promote cell apoptosis. **A** The histogram showed that the mRNA level GBP1 gene knockdown experiment was successful. **B** The knock-down effect of GBP1 was verified by western blot. **C** The proliferation of tumor cells after GBP1 knockdown was detected by CCK-8 assay. **D** Cell invasion experiment after GBP1 knockdown. **E** The apoptosis of GBP1 knockdown cells was detected by flow cytometry. Repeat 3 times per set. GAPDH as the internal reference. *siRNA* small interfering RNA, *si-NC* negative control siRNA, *si-GBP1* GBP1 siRNA, *GAPDH* glyceraldehyde-3-phosphate dehydrogenase. *p < 0.05, **p < 0.01

Based on the above GBP1 may be related to the poor prognosis of patients with cervical cancer, in order to further study the effect of GBP1 on cervical cancer cells, we conducted GBP1 knockdown assay. In Caski cells, GBP1 gene knockdown, qPCR detection of knockdown effect reached more than 70%, mRNA level knockdown successfully (p < 0.0001) (Fig. 9A). Endogenous antibody was used for Westernblot to detect the target band of the corresponding protein size (about 68kDa), which had a knock-down effect at the protein level (t = 7.273, p = 0.0019) (Fig. 9B). CCK8 results showed that GBP1 silences inhibited cell proliferation from 48 to 72 h (t_48h_ = 4.309, p_48h_ = 0.0126; t_72h_ = 7.795, P_72h_ = 0.0015) (Fig. 9C). Cell invasion results showed that GBP1 silencing inhibited cell invasion compared with the control group (t = 3.876, p = 0.0179) (Fig. 9D). Apoptosis results showed that GBP1 silenced promoted apoptosis (t = 6.370, p = 0.0031) (Fig. 9E).

### GBP1 overexpression can promote the proliferation and invasion of cervical cancer cells

**Fig. 10:**
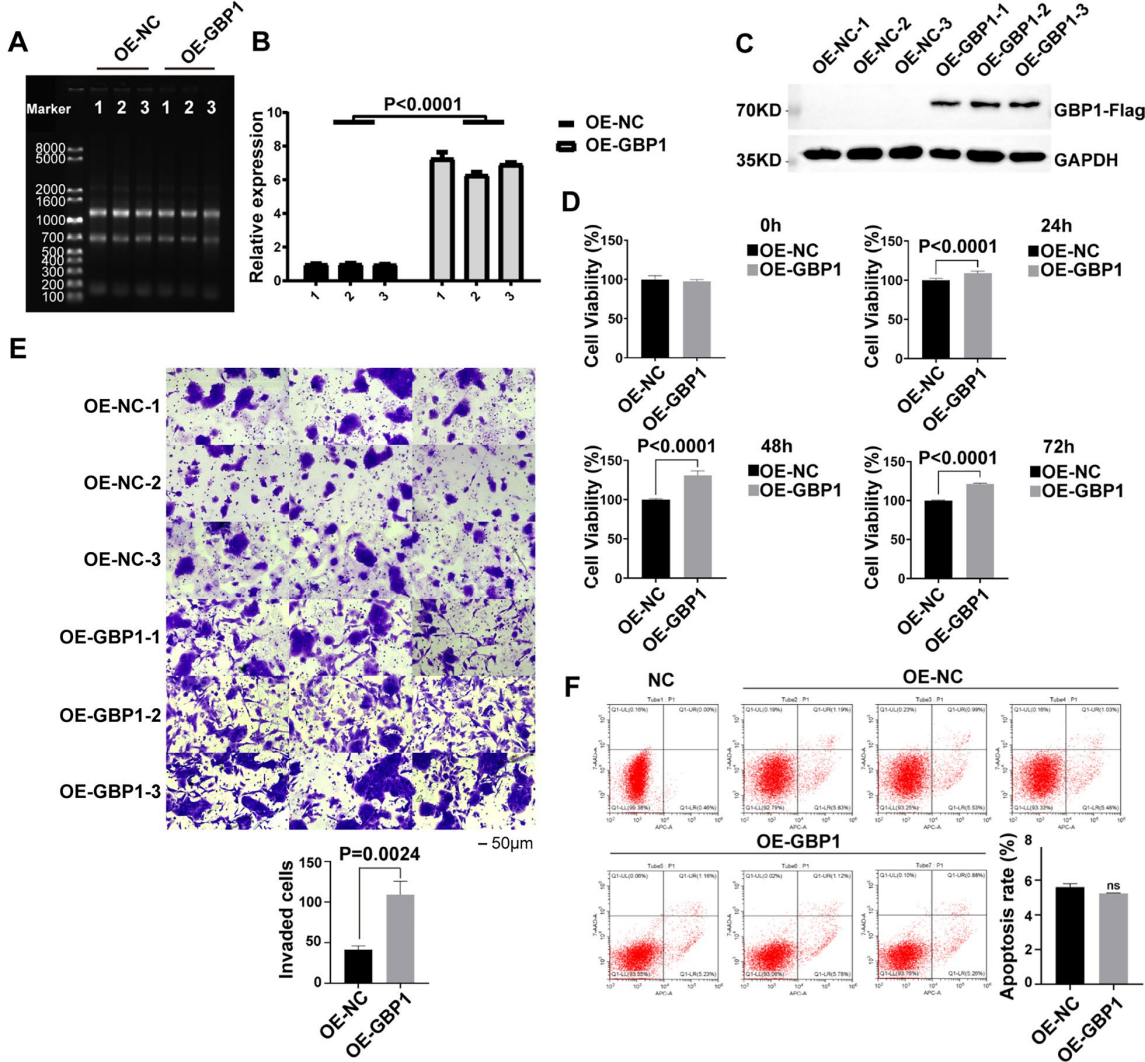
GBP1 overexpression can promote the proliferation and invasion of cervical cancer cells. **A** Agarose gel electrophoresis showed successful RNA extraction. **B** RT-qPCR showed that GBP1 mRNA was overexpressed successfully. **C** Western blotting showed that GBP1 protein was overexpressed successfully. **D** The proliferation of tumor cells after GBP1 overexpression was detected by CCK-8 assay. **E** Cell invasion experiment after GBP1 overexpression. **F** The apoptosis of GBP1 overexpression cells was detected by flow cytometry. Repeat 3 times per set. GAPDH as the internal reference. *OE* overexpression, *NC* negative control, *GAPDH* glyceraldehyde-3-phosphate dehydrogenase

After transfecting Caski cells with GBP1 overexpression vector, RNA was extracted. Agarose gel electrophoresis showed that RNA was extracted successfully. RT-qPCR results showed that the mRNA level was overexpressed 6–7 times, and in the cell model of Caski cell line overexpressing GBP1 gene, the mRNA level was successfully overexpressed (Fig. 10A, B). The molecular weight of GBP1 is 68KD, while the actual detected molecular weight of westernblot is 70KD. According to the pictures of westernblot, bands were detected in OE-GBP1 samples, while no bands were detected in OE-NC samples. This indicated that GBP1 was successfully overexpressed at the protein level (Fig. 10C). Subsequently, cell proliferation was measured by CCK8 assay. The results showed that GBP1 overexpression promoted cell proliferation at 24 h to 72 h (t_24h_ = 6.249, t_48h_ = 12.99, t_72h_ = 36.15, p_24h,48h,72h_ < 0.0001) (Fig. 10D). Cell invasion results showed that overexpression of GBP1 promoted cell invasion compared with the control group (t = 6.843, p = 0.0024) (Fig. 10E). Apoptosis results showed that overexpression of GBP1 inhibited apoptosis, but there was no significance of T-test (t = 0.7383, p = 0.5013) (Fig. 10F).

### GBP1 overexpression in cervical cancer can promote tumor growth in vivo

**Fig. 11:**
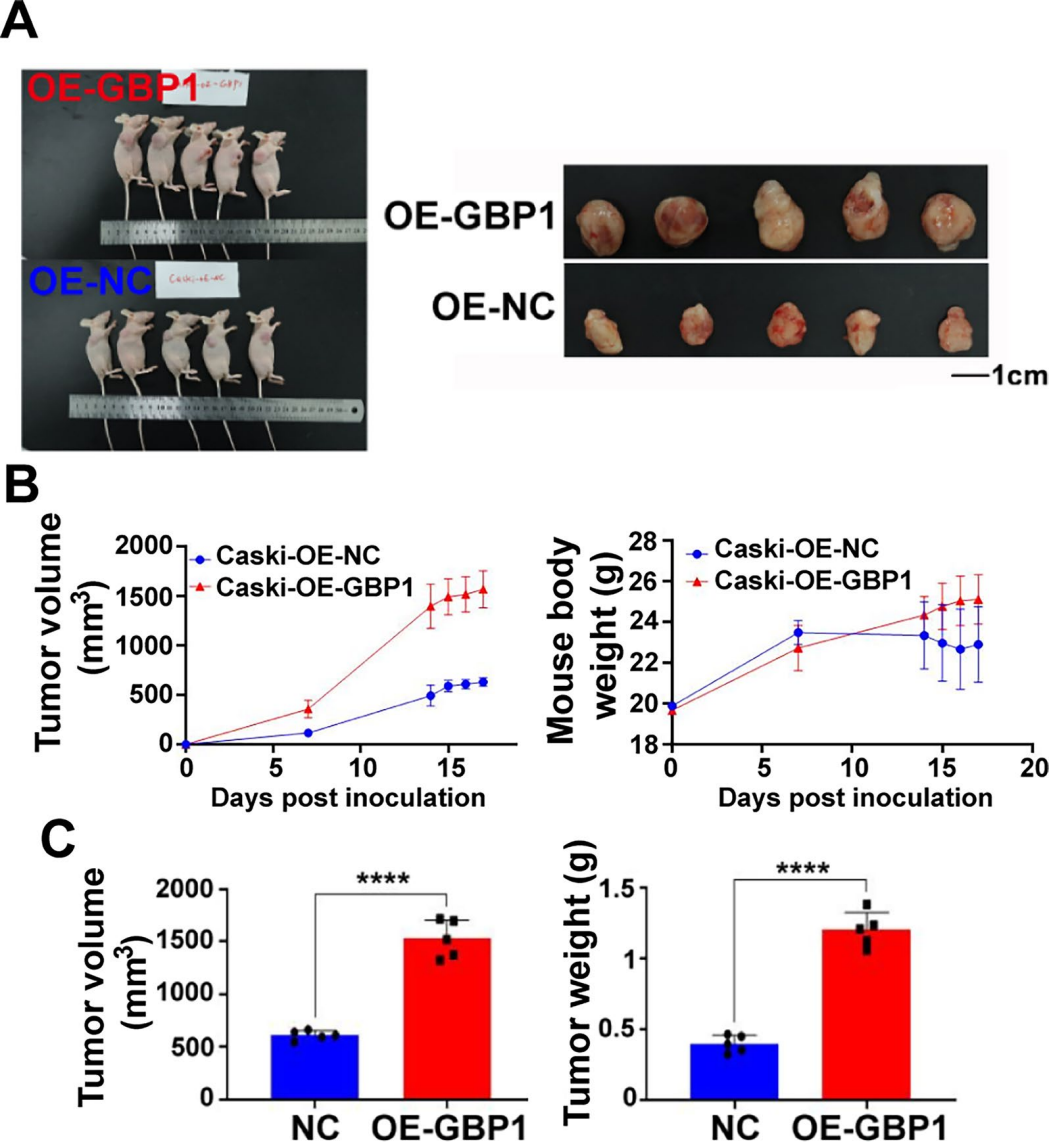
Animal experiment showed that GBP1 overexpression could promote tumor growth. **A** Animal models after 17 days. **B** Tumor growth curve of Caski nude mice. **C** The tumor size and weight were compared between Caski nude mice and control group after 17 days. *OE* overexpression, *NC* negative control. ****p < 0.0001

In vivo experiment, 5 nude mice were taken and subcutaneously injected with Caski cells overexpressing GBP1 and negative control cells. The weight and tumor volume of mice were measured at days 0, 7, 14, 15, 16 and 17, and the results showed that the final tumor volume and weight of mice in the GBP1 overexpression group were significantly increased compared with the negative control group (t_1_ = 11.10, t_2_ = 13.26, p < 0.0001) (Fig. 11A–C).

### GBP1 is associated with a large number of alternative splicing events

**Fig. 12:**
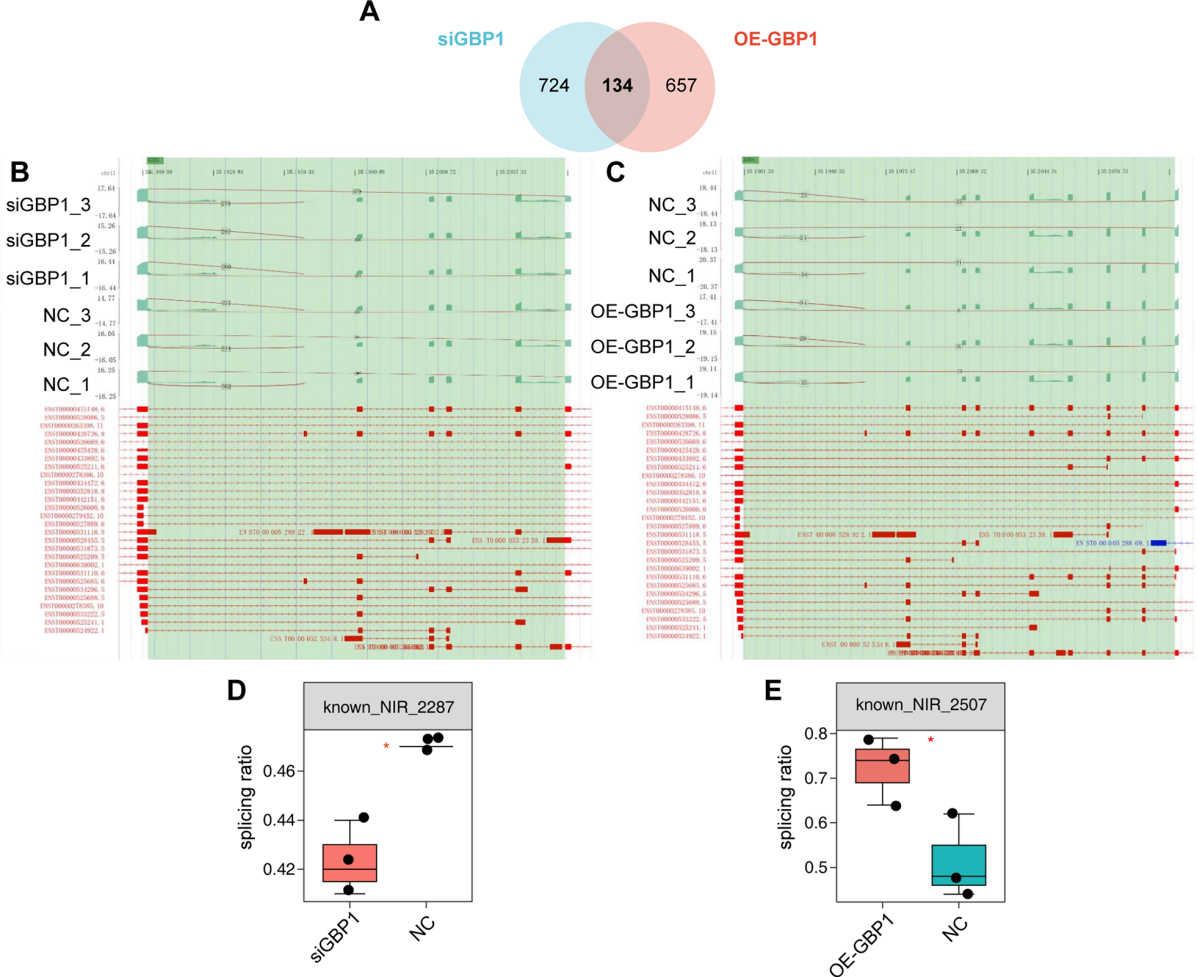
GBP1 enhances CD44 splicing ratio. **A** The Venn diagram showed the intersection of variable splice-related genes after GBP1 knockdown and overexpression. **B**, **C** Fishbone diagrams show the A3SS variable splicing events of CD44 after GBP1 knockdown and overexpression. **D**, **E** Changes in splicing ratio of A3SS variable splicing events of CD44 after GBP1 knockdown and overexpression. *siGBP1* GBP1 siRNA, *OE* overexpression, *NC* negative control

GBP1 overexpression regulates 988 alternative splicing sites, involving 791 genes. The enrichment analysis of these 791 genes showed that they were significantly enriched in MFs such as protein/zinc ion/ATP/DNA binding, significantly enriched in CCs such as nucleoplasma, cytoplasm and nucleus, and significantly enriched in BPs such as DNA repair, positive regulation of I-kappaB kinase/NF-kappaB cascade, and transcriptional regulation (Additional file 2: Figure S2A). KEGG enrichment analysis showed significant enrichment in pyrimidine/purine metabolism and toll-like receptor signaling pathways (Additional file 2: Figure S2B). GBP1 knock-down expression regulates 1052 alternative splicing sites, involving 858 genes. Enrichment analysis was performed on the 858 genes. GO enrichment analysis showed that MFs such as protein/RNA/phosphatidylinositol-3,4,5-triphosphate binding and methyltransferase activity were significantly enriched, and CCs such as nucleus, nucleoplasma and cytoplasm were significantly enriched. Significantly enriched in BPs such as gene expression, histone methylation, Notch signaling pathway (Additional file 2: Figure S2C); KEGG enrichment analysis showed significant enrichment in ubiquitin-mediated proteolysis, Hippo signaling, platinum resistance and other pathways (Additional file 2: Figure S2D). When the two were intersected, 134 genes were involved in total (Fig. 12A). After analysis of 134 genes, it was found that the same base site of CD44 in GBP1 overexpression and inhibited expression occurred alternative 3ʹ splice site (A3SS) variable splicing event (Fig. 12B, C). After GBP1 silencing, the splicing rate was significantly lower than that of the control group, and the opposite was true after GBP1 overexpression (p < 0.05) (Fig. 12D, E). GBP1 can regulate the splicing changes of CD44 and improve the splicing rate of CD44.

### GBP1 is not a alternative splicing factor

**Fig. 13:**
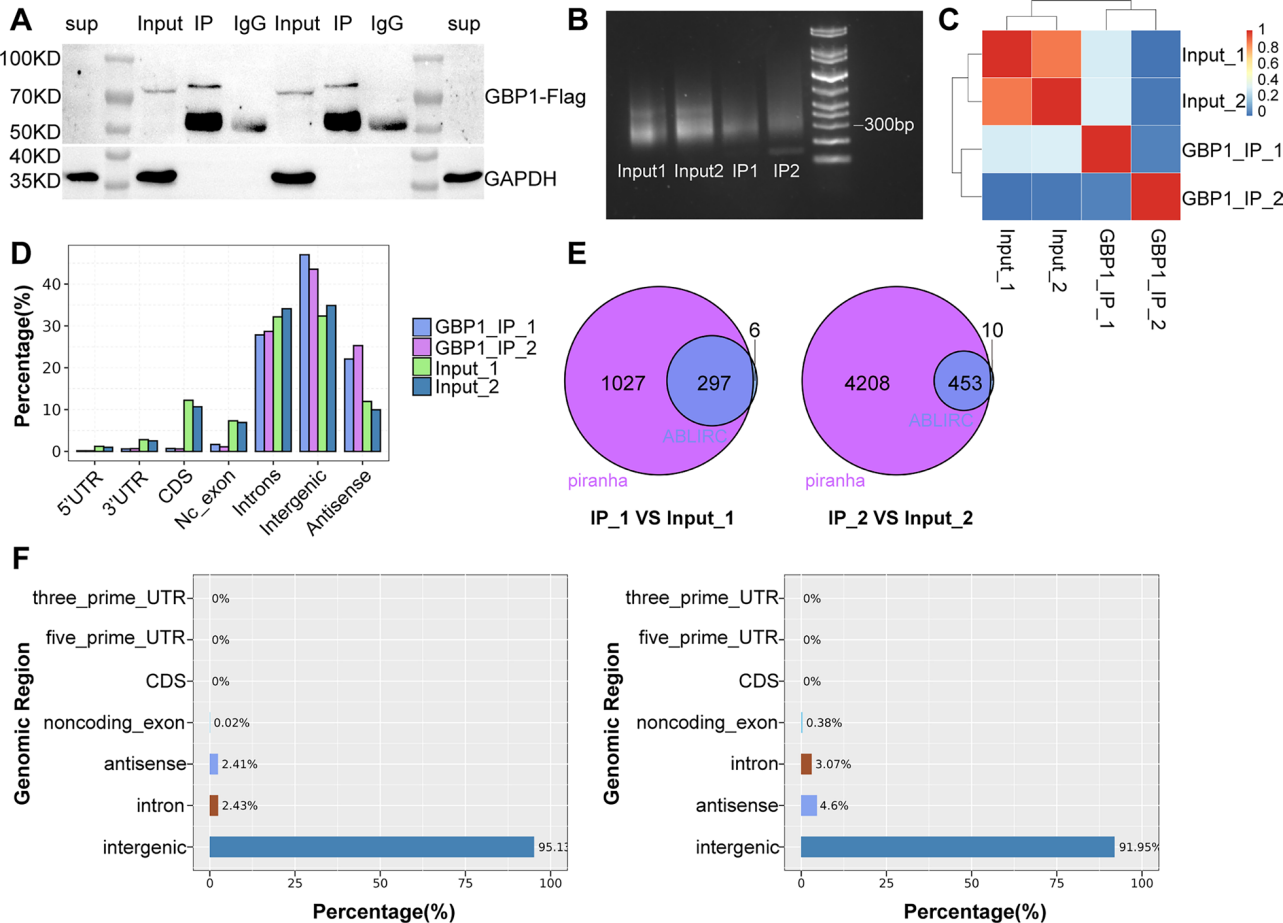
The iRIP-seq results of GBP1 in Caski cells. **A** Western blot test of IP efficiency. 70 kDa is the estimated size of GBP1 molecule. **B** IP strip of the Sequence. **C** Heat map showing the hierarchically clustered Pearson correlation matrix resulting from comparing the transcript expression values for control and GBP1 IP samples. **D** Bar plot of the genomic region distribution of the control and uniquely mapped GBP1 IP reads. **E** Venn diagram analysis from the comparative result of ABLIRC and Piranha peak calling methods. **F** The peak distribution across reference genomic region of the GBP1 IP and control group. *iRIP-seq* improved RNA immunoprecipitation sequencing, *IP* immunoprecipitation, *Input* blank control, *IgG* negative control

To the best of our knowledge, classical RNA Binding Protein (RBP) is evaluated by its binding power and combined read volume as well as efficiency. For these reasons, we attempted to explore the binding capacity of GBP1 using RNA immunoprecipitation (RIP). We used improved RIP and high-throughput sequencing methods (iRIP-seq) to identify transcripts interacting with GBP1 in Caski cells. Westernblot showed good IP efficiency (Fig. 13A). Unfortunately, the result showed that GBP1 directly interacted with very few transcripts and had very few events in the CDS, 5ʹ UTR, and 3ʹ UTR regions, proving that GBP1 is not a classical RBP and is unlikely to be a alternative splicing factor. We believe that a large number of AS events regulated by it may be conducted through indirect channels (Fig. 13B–F).

### Prediction and enrichment analysis of GBP1 interacting proteins

**Fig. 14:**
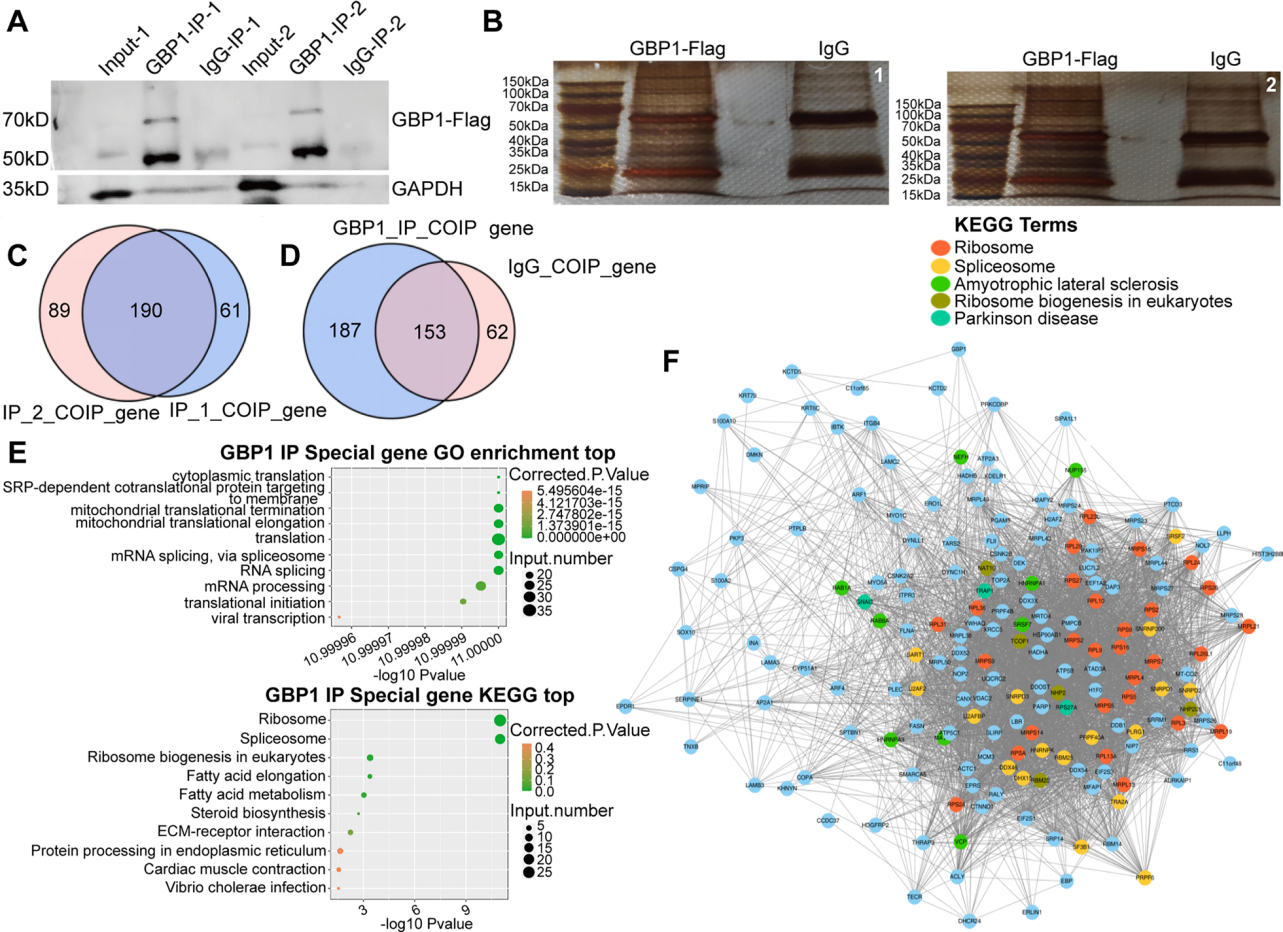
The CoIP-MS results of GBP1 in Caski cells. **A** The results of CoIP were verified by western blotting. Input group is the blank control group, showing background level, and IgG-IP group is the negative control. GAPDH was chosen as the internal reference. 70 kDa is the estimated size of GBP1 molecule. **B** Silver staining was used to separate mixtures bound to GBP1. 1 and 2 represented two repeated experiments. **C** Venn diagram showing the overlapped and specific GBP1-interacted proteins between IP_1 and IP_2 samples. **D** Venn diagram showing the overlapped and specific GBP1-interacted proteins between IP and IgG samples. (187 GBP1-specific interacting proteins). **E** Bubble plot showing the top ten enriched GO BP pathways and KEGG pathways for GBP1 specific interacted proteins. **F** Network plot showing the relationship of proteins specific interacted with GBP1. *CoIP-MS* co-immunoprecipitation-mass spectroscopy, *IP* immunoprecipitation

Based on the results of previous iRIP-seq assays, we conducted CoIP-MS assays on GBP1 in Caski cells. After CoIP test, Flag and IgG antibodies were used to precipitate the proteins. In westernblot, Input group was the control group, showing background level, IgG-IP group was the negative control. The results showed that in the two repeated experiments, GBP1-IP group had obvious bands at 70kDa, consistent with the expected size of GBP1, but not in the Input group and IgG-IP group (Fig. 14A). Silver staining showed significant differences in the bands between GBP1-Flag group and IgG group, indicating the presence of GBP1-specific binding proteins (Fig. 14B). Westernblot and silver staining confirmed the success of CoIP. According to the results of our CoIP-MS experiment, all identified interacting proteins (n = 865) were firstly analyzed by GO, KEGG, COG and IPR enrichment, and the subcellular localization of these proteins was conducted. The results showed that these proteins were mainly enriched in translation and other BPs, ribosome and other CCs, and MFs such as protein binding in GO terms (Additional file 3: Figure S3A). KEGG enrichment analysis showed that these proteins were mainly enriched in KEGG pathways such as translation and viral infectious diseases (Additional file 3: Figure S3B). In addition, these proteins were mainly concentrated in the COG function of class J (translation, ribosomal structure and biogenesis) (Additional file 3: Figure S3C) and IPR annotations such as immunoglobulin V-set domain, immunoglobulin subtype, and immunoglobulin-like domain (Additional file 3: Figure S3D). Subcellular localization showed that these proteins were mainly nucleus proteins (Additional file 3: Figure S3E). Among these proteins, a total of 348 proteins were enriched in GO, KEGG, COG and IPR annotations (Additional file 3: Figure S3F). In our experimental results, a total of 190 binding proteins were detected in the GBP1-IP group in the two repeated experiments (Fig. 14C), including this portion, were detected in both the GBP1-IP group and IgG-IP group. Similarly, the 865 proteins also contained the part that could be detected in the IgG-IP group but not in the GBP1-IP group.

**Fig. 15:**
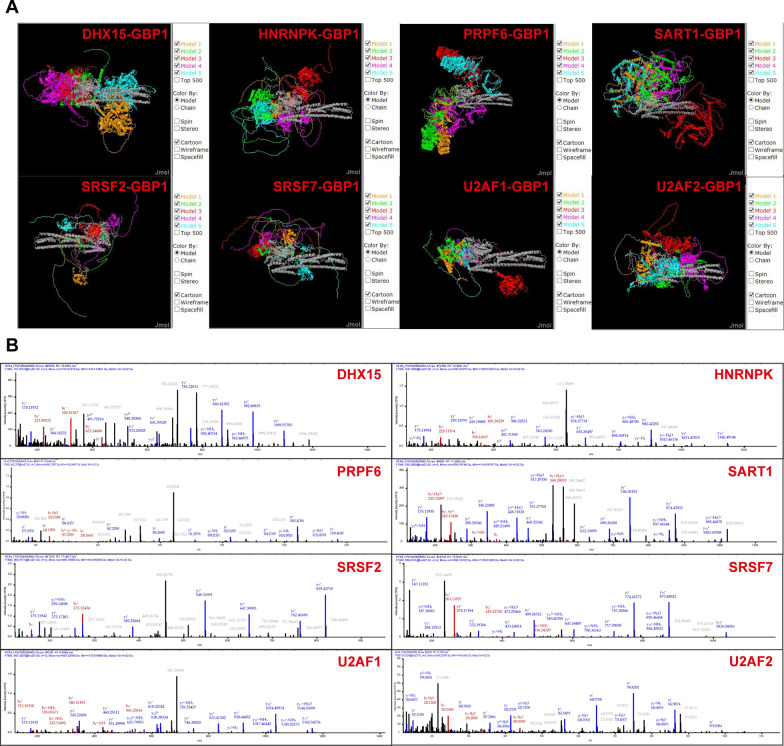
CoIP-MS representative diagrams. **A** The ZDOCK protein interaction analysis of GBP1-interacted alternative splicing factor. (GBP1 is described in gray. The top ten possible binding models were selected for each alternative splicing factor and GBP1). **B** Representative peptide mass chromatogram of eight splicing factors binding to GBP1 detected by COIP-MS. (Total including 15 DHX15 peptides, 27 HNRNPK peptides, 2 PRPF6 peptides, 3 SART1 peptides, 9 SRSF2 peptides, 3 SRSF7 peptides, 1 U2AF1 peptide and 6 U2AF2 peptides)

**Fig. 16:**
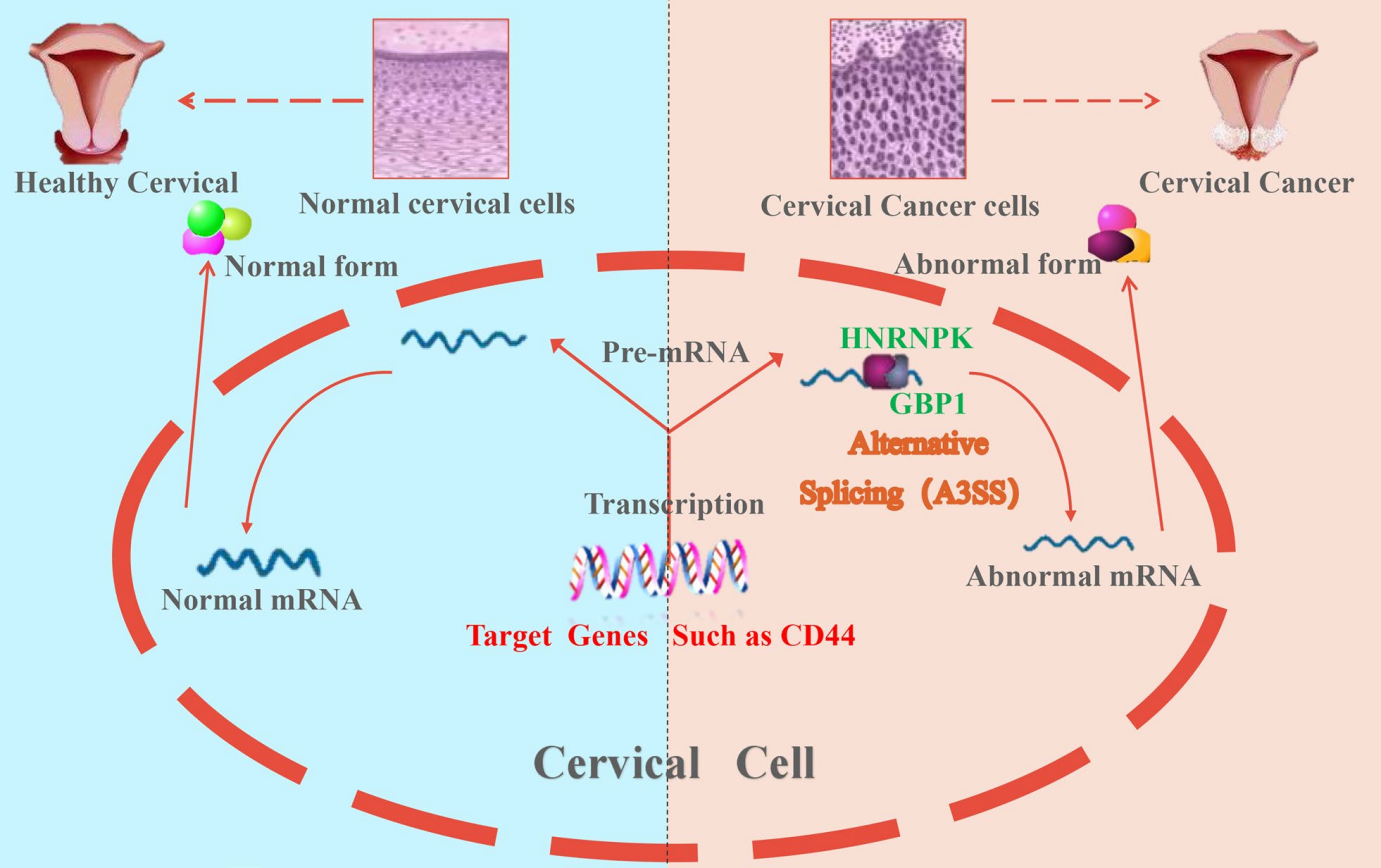
A potential pathway of promoting cancer in cervical cancer (Binding GBP1 to HNRNPK through A3SS pathway to regulate CD44 alternative splicing)

After screening, 187 proteins specifically binding to GBP1 were finally found in Caski cell line (Fig. 14D), as detailed in Additional file 4: Table S1. Subsequently, the 187 GBP1 interacting proteins were enriched and analyzed. GO analysis showed that GBP1 interacting protein was involved in RNA splicing. According to KEGG analysis, 17 of these 187 proteins were found to be functionally enriched into spliceosome (Fig. 14E). The PPI networks of 187 GBP1-specific interacting proteins were shown in Fig. 14F, and the KEGG term of each protein was labeled. Spliceosome includes reported alternative splicing factors such as DHX15, Heterogeneous Nuclear Ribonucleoprotein K (HNRNPK), pre-mRNA processing factor 6 (PRPF6), SQUAMOUS CELL CARCINOMA ANTIGEN RECOGNIZED BY T CELLS 1(SART1), Serine/arginine-rich splicing factor 2 (SRSF2), Serine/arginine-rich splicing factor 7 (SRSF7), U2 small nuclear RNA auxiliary factor 1 (U2AF1) and U2 small nuclear RNA auxiliary factor 2 (U2AF2) (Fig. 15). In combination with existing reports [24] and GBP1-regulated AS events, a regulatory pathway was finally obtained, that is, GBP1 regulated CD44 protein expression through A3SS alternative splicing after binding to HNRNPK, and finally played a role in promoting cancer (Fig. 16).

## Discussion

Cervical cancer, the fourth most common cancer in women, has a higher mortality rate in developing and underdeveloped countries [1]. GBP1, as an important member of the GBP family, has the functions of resisting various viruses, bacterias and protozoas [4–6], anti-angiogenesis effect [7], and even anti-tumor effect in some cancers [12]. GBP1 has been reported to be associated with better prognosis in colorectal cancer, liver cancer, epithelial ovarian cancer, triple negative breast cancer, cutaneous melanoma and other cancers [12–14, 25–27]. And many studies showed that it was associated with tumor cell growth, invasion and metastasis in lung cancer, glioblastoma multiforme, esophageal squamous cell carcinoma, oral squamous cell carcinoma, head and neck carcinoma and low-grade glioma, and predicted a poor prognosis [17–19, 28–30]. However, there is a lack of studies on cervical cancer. Therefore, we conducted in-depth studies on the function and mechanism of GBP1 in the occurrence and progression of cervical cancer through bioinformatics, multicolor immunofluorescence staining, knockdown and overexpression assays, building animal models, CCK-8 detection, cell invasion assay, apoptosis detection, alternative splicing analysis, iRIP-seq and CoIP-MS, and other methods. To explore its significance in cervical cancer and its possibility as a new tumor marker for cervical cancer. Finally, a regulatory pathway was obtained. We believe that GBP1 and HNRNPK bind to regulate the expression of CD44 protein through A3SS alternative splicing form, and finally play a role in promoting cancer.

In this study, we first found that GBP1 was widely present in a variety of tumor tissues and immune cells, including cervical cancer tissues. Our gene correlation analysis included the correlation between GBP1 and miRNAs, lncRNAs, mutated genes and protein-coding genes. GBP1 was found to be the target gene of miR-377-3p, miR-335-5p, miR-944, miR-543, and miR-532-5p, and was significantly correlated with lncrnas such as LINC02195, LINC02446, LINC02528 (Cor ≥ 0.6). In CESC, mutations in MYH9, SPEN, MUC17, KRAS and ZNF750 genes affected the expression level of GBP1. GBP1 was significantly positively correlated with genes such as GBP4, CXCL10, TAP1, STAT1 and GBP1P1 (Cor ≥ 0.7), and negatively correlated with genes such as SNORC, VPS37D, SPINK1, HES6 and CDHR3 (Cor ≤ -0.4). At the same time, we found that the high expression of GBP1 in cervical cancer was related to drug sensitivity such as Cediranib, BLU-667, JNJ-42756493 and Pazopanib, which is manifested as treatment resistance.

GBP1 has been reported to play an important role in tumor immunity. For example, De Buhr et al. [31] identified GBP1 as one of the leading candidate genes that play an important role in inflammatory processes and immune responses. Mustafa et al. [32] suggested that GBP1 is a key induction proteins of T lymphocytes, and T lymphocytes affect tumor metastasis by inducing GBP1 expression. Britzen-laurent et al. [12] suggested that the loss of GBP1 expression in colorectal cancer suggests that the tumor evades the Th1 immune response dominated by IFN-γ, and GBP1 is involved in the anti-tumor immune response. Similarly, our study found that GBP1 played an important role in tumor immunity in cervical cancer. The tumor microenvironment often seriously affects the genesis and progression of tumor. In the tumor immunocorrelation analysis of GBP1, we found that GBP1 was significantly associated with a large number of immunoinfiltrating cells. Among them, we found that GBP1 was the most correlated with CD8+ T cells, CD4+ T cells, macrophages, neutrophils, MDCs, Tcm CD8, γδ Tcells and NK cells. In addition, we found that GBP1 had a strong positive correlation with immune score and tumor microenvironment score, indicating that GBP1 expression is significantly correlated with tumor microenvironment, and plays an important role in the occurrence and development of tumors. At the same time, we found that GBP1 expression was significantly positively correlated with immunosuppressants CD274, CD96, CSF1R, CTLA4, HAVCR2, IDO1, LAG3, PDCD1, PDCD1LG2, and TIGIT (Cor ≥ 0.6). It was positively correlated with immunostimulants CD48, CD80, CD86, ICOS, IL2RA, KLRK1, LTA, TNFRSF9, TNFSF13B (Cor ≥ 0.6). Based on the correlation analysis of these immune checkpoint genes, the discovery of new immune checkpoint inhibitors or stimulants is beneficial to the treatment of patients with cervical cancer [33]. In addition, GBP1 expression was significantly positively correlated with chemokines such as CCL4, CCL5, CCL8, CXCL9, CXCL10, CXCL11, CXCL13 (Cor ≥ 0.6), and chemokine receptors such as CCR1, CCR5, CXCR3, CXCR6 (Cor ≥ 0.6). Subsequently, based on bioinformatics analysis, GBP1-related genes and differentially expressed genes were enriched and found to be involved in many immune pathways, such as T cell activation, signaling pathway activation, cytokine regulation, IFN-γ signaling pathway, and the interaction between immune cells and miRNA in the tumor microenvironment.

The common PD-1/PD-L1 pathway interaction occurs when PD-1 expressed on activated T cells interacts with PD-L1 on tumor cells, resulting in suppression of T cell activation, enabling immune escape. PD-L1 is also expressed on the surface of some tumor infiltrating lymphocytes [34], and there may be cis-interaction with PD-1 on the surface of T cells. In order to explore the influence and mechanism of GBP1 on the occurrence and progression of cervical cancer, we analyzed the correlation between GBP1 and PD-1 and PD-L1 in cervical cancer tissues by multicolor immunofluorescence staining. We found that the expression of GBP1 on TILs was strongly correlated with the expression of PD-1 and PD-L1, and the expression of GBP1 on cervical cancer cells was strongly correlated with the expression of PD-L1. In addition, we found that the expression of GBP1 on cervical cancer cells was also correlated with the expression of PD-1 and PD-L1 on TILs. The prognostic analysis of 104 cases of cervical cancer showed that high GBP1 level on tumor cells was associated with poor prognosis of cervical cancer patients. At the same time, multivariate analysis showed that it could be used as an independent prognostic factor. For the first time, we had linked GBP1 to cancer-promoting effects in cervical cancer.

To further explore the effect of GBP1 on the occurrence and progression of cervical cancer. We performed knockdown and overexpression experiments on GBP1, and validated it in vivo in animal models. We found that GBP1 silencing could inhibit the proliferation and invasion of cervical cancer cells and promote the apoptosis of tumor cells. Meanwhile, we found that GBP1 overexpression could promote the proliferation and invasion of cervical cancer cells, and GBP1 overexpression could promote tumor growth in cervical cancer. Therefore, we believe that GBP1 has a cancer-promoting effect in cervical cancer. In order to further study the mechanism of the cancer-promoting effect of GBP1, we first conducted a alternative splicing analysis of GBP1 overexpression and suppressed expression, involving a total of 134 AS-related genes, and found that CD44 had A3SS alternative splicing events at the same base site in GBP1 overexpression and silencing. We believe that GBP1 can regulate the splicing changes of CD44 and improve the splicing rate of CD44. However, in our further study, the iRIP-seq results showed that GBP1 was not a classical RBP, in other words, GBP1 might not be a alternative splicing factor, and a large number of AS events regulated by GBP1 might be conducted through indirect pathways. In order to further investigate the cancer-promoting mechanism of GBP1, we tried to find a reliable pathway. In our CoIP-MS experiment, 187 GBP1-specific interacting proteins were obtained. Among them, a total of 17 proteins were functionally enriched in spliceosome, including DHX15, HNRNPK, PRPF6, SART1, SRSF2, SRSF7, U2AF1 and U2AF2, which have been reported as alternative splicing factors. Combined with the existing reports [24] and GBP1-regulated AS events, we finally obtained a regulatory pathway, that is, GBP1 and HNRNPK binding, through the A3SS alternative splicing form, regulate the expression of CD44 protein, and finally play a cancer-promoting role in cervical cancer.

In summary, to the best of our knowledge, this study was the first to examine in depth the effects of GBP1 in cervical cancer. This study focused on the important role of GBP1 in tumor immunity, its cancer-promoting effect on cervical cancer and its mechanism. For example, GBP1 increases the tolerance of tumor cells to some drugs and has a strong correlation with the PD-1/PD-L1 pathway. We found that GBP1 silencing could inhibit the proliferation and invasion of cervical cancer cells and promote the apoptosis of tumor cells. GBP1 overexpression can promote the proliferation and invasion of cervical cancer cells and promote tumor growth. More importantly, we found that GBP1 could improve the splicing rate of CD44. However, our experiment found that GBP1 was not a alternative splicing factor, so it was speculated that it plays a cancer-promoting role by binding with the true alternative splicing factors, and indirectly participates in the alternative splicing pathway. HNRNPK was discovered as one of the alternative splicing factors combined with GBP1. Finally, we proposed a meaningful model for promoting cancer. That is, GBP1, after binding with HNRNPK, regulates the expression of CD44 protein through A3SS alternative splicing form, and finally plays a cancer-promoting role in cervical cancer. The shortcoming of this study is the lack of direct verification of GBP1-HNRNPK-CD44 pathway. In the future, we will verify the cancer-promoting effect of this pathway in cervical cancer through various biological experiments.

### Supplementary Information

**Additional file 1: Figure S1.** Correlation between GBP1 expression and infiltrating immune cells in MCP-counter and quanTIseq algorithms.

**Additional file 2: Figure S2.** AS event-related gene enrichment analysis. **(A)** GO analysis of AS event-related genes after GBP1 overexpression. **(B)** KEGG analysis of AS event-related genes after GBP1 overexpression. **(C)** GO analysis of AS event-related genes after GBP1 expression inhibition. **(D)** KEGG analysis of AS event-related genes after GBP1 expression inhibition. AS: alternative splicing, MF: molecular function, CC: cellular component, BP: biological process.

**Additional file 3: Figure S3.** Enrichment analysis of CoIP-MS original results, including GBP1-IP group detected (not detected in IgG-IP group), IgG-IP group detected (not detected in GBP1-IP group) and two groups detected. **(A)** GO annotation of interacting proteins in terms of biological processes, cellular components, and molecular functions. **(B)** KEGG analysis of interacting proteins. **(C)** COG function classification of interacting protein pathways. **(D)** IPR analysis of interacting proteins. **(E)** Subcellular localization of interacting proteins. **(F)** Venn diagram showed the number of proteins annotated by GO, KEGG, COG and IPR.

**Additional file 4: Table S1.** 187 GBP1-specific interacting proteins.

## Data Availability

The data that support the findings of this study are available on request from the corresponding author.

## References

[1] Arbyn M, Weiderpass E, Bruni L (2020). Estimates of incidence and mortality of cervical cancer in 2018: a worldwide analysis. Lancet Glob Health.

[2] Tabibi T, Barnes JM, Shah A (2022). Human papillomavirus vaccination and trends in cervical cancer incidence and mortality in the US. JAMA Pediatr.

[3] Cheng YS, Colonno RJ, Yin FH (1983). Interferon induction of fibroblast proteins with guanylate binding activity. J Biol Chem.

[4] Glitscher M, Himmelsbach K, Woytinek K (2021). Identification of the interferon-inducible GTPase GBP1 as major restriction factor for the Hepatitis E virus. J Virol.

[5] Kutsch M, Sistemich L, Lesser CF (2020). Direct binding of polymeric GBP1 to LPS disrupts bacterial cell envelope functions. Embo j.

[6] Fisch D, Clough B, Domart MC (2020). Human GBP1 differentially targets salmonella and toxoplasma to license recognition of microbial ligands and caspase-mediated death. Cell Rep.

[7] Weinländer K, Naschberger E, Lehmann MH (2008). Guanylate binding protein-1 inhibits spreading and migration of endothelial cells through induction of integrin alpha4 expression. FASEB J.

[8] Britzen-Laurent N, Herrmann C, Naschberger E (2016). Pathophysiological role of guanylate-binding proteins in gastrointestinal diseases. World J Gastroenterol.

[9] Persano L, Moserle L, Esposito G (2009). Interferon-alpha counteracts the angiogenic switch and reduces tumor cell proliferation in a spontaneous model of prostatic cancer. Carcinogenesis.

[10] Lipnik K, Naschberger E, Gonin-Laurent N (2010). Interferon gamma-induced human guanylate binding protein 1 inhibits mammary tumor growth in mice. Mol Med.

[11] Unterer B, Wiesmann V, Gunasekaran M (2018). IFN-γ-response mediator GBP-1 represses human cell proliferation by inhibiting the Hippo signaling transcription factor TEAD. Biochem J.

[12] Britzen-Laurent N, Lipnik K, Ocker M (2013). GBP-1 acts as a tumor suppressor in colorectal cancer cells. Carcinogenesis.

[13] Takagi K, Takayama T, Nagase H (2011). High TSC22D3 and low GBP1 expression in the liver is a risk factor for early recurrence of hepatocellular carcinoma. Exp Ther Med.

[14] Carbotti G, Petretto A, Naschberger E (2020). Cytokine-Induced Guanylate Binding Protein 1 (GBP1) release from human ovarian cancer cells. Cancers (Basel)..

[15] Wu Y, Xia L, Zhao P (2020). Immune profiling reveals prognostic genes in high-grade serous ovarian cancer. Aging (Albany NY).

[16] Cheng L, Gou L, Wei T (2020). GBP1 promotes erlotinib resistance via PGK1-activated EMT signaling in non-small cell lung cancer. Int J Oncol.

[17] Meng Y, Wang W, Chen M (2020). GBP1 facilitates indoleamine 2,3-dioxygenase extracellular secretion to promote the malignant progression of lung cancer. Front Immunol.

[18] Ji X, Zhu H, Dai X (2019). Overexpression of GBP1 predicts poor prognosis and promotes tumor growth in human glioblastoma multiforme. Cancer Biomark.

[19] Li L, Ma G, Jing C (2015). Guanylate-binding protein 1 (GBP1) promotes lymph node metastasis in human esophageal squamous cell carcinoma. Discov Med.

[20] Livak KJ, Schmittgen TD (2001). Analysis of relative gene expression data using real-time quantitative PCR and the 2(-Delta Delta C(T)) Method. Methods.

[21] Kim D, Pertea G, Trapnell C (2013). TopHat2: accurate alignment of transcriptomes in the presence of insertions, deletions and gene fusions. Genome Biol.

[22] Xia H, Chen D, Wu Q (2017). CELF1 preferentially binds to exon-intron boundary and regulates alternative splicing in HeLa cells. Biochim Biophys Acta Gene Regul Mech.

[23] Uren PJ, Bahrami-Samani E, Burns SC (2012). Site identification in high-throughput RNA-protein interaction data. Bioinformatics.

[24] Peng WZ, Liu JX, Li CF (2019). hnRNPK promotes gastric tumorigenesis through regulating CD44E alternative splicing. Cancer Cell Int.

[25] Criscitiello C, Bayar MA, Curigliano G (2018). A gene signature to predict high tumor-infiltrating lymphocytes after neoadjuvant chemotherapy and outcome in patients with triple-negative breast cancer. Ann Oncol.

[26] Wang Q, Wang X, Liang Q (2018). Distinct prognostic value of mRNA expression of guanylate-binding protein genes in skin cutaneous melanoma. Oncol Lett.

[27] Pedersen MH, Hood BL, Beck HC (2017). Downregulation of antigen presentation-associated pathway proteins is linked to poor outcome in triple-negative breast cancer patient tumors. Oncoimmunology.

[28] Yu CJ, Chang KP, Chang YJ (2011). Identification of guanylate-binding protein 1 as a potential oral cancer marker involved in cell invasion using omics-based analysis. J Proteome Res.

[29] Wu ZH, Cai F, Zhong Y (2020). Comprehensive analysis of the expression and prognosis for GBPs in Head and neck squamous cell carcinoma. Sci Rep.

[30] Johnson H, Del Rosario AM, Bryson BD (2012). Molecular characterization of EGFR and EGFRvIII signaling networks in human glioblastoma tumor xenografts. Mol Cell Proteomics.

[31] de Buhr MF, Mähler M, Geffers R (2006). Cd14, Gbp1, and Pla2g2a: three major candidate genes for experimental IBD identified by combining QTL and microarray analyses. Physiol Genomics.

[32] Mustafa DAM, Pedrosa R, Smid M (2018). T lymphocytes facilitate brain metastasis of breast cancer by inducing Guanylate-Binding Protein 1 expression. Acta Neuropathol.

[33] O'Malley DM, Neffa M, Monk BJ (2022). Dual PD-1 and CTLA-4 checkpoint blockade using balstilimab and zalifrelimab combination as second-line treatment for advanced cervical cancer: an open-label phase II study. J Clin Oncol.

[34] Thike AA, Chen X, Koh VCY (2020). Higher densities of tumour-infiltrating lymphocytes and CD4(+) T cells predict recurrence and progression of ductal carcinoma in situ of the breast. Histopathology.

