# Supplementary figures and images for: Function and mechanism of GBP1 in the development and progression of cervical cancer
Source: J Transl Med. 2024 Jan 2;22:11. doi: 10.1186/s12967-023-04837-6 (PMC10763113; doi:10.1186/s12967-023-04837-6)

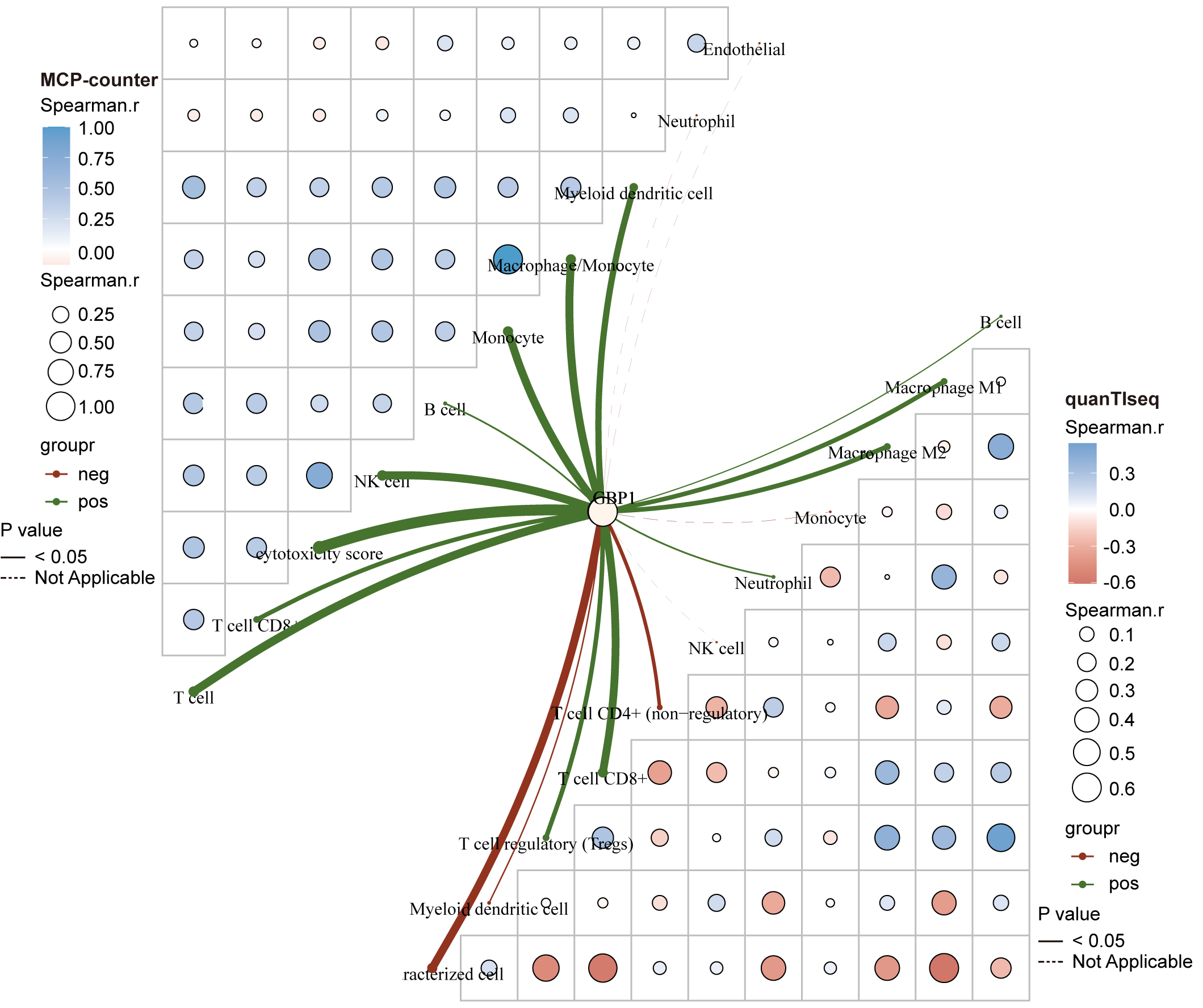

Supplement: Supplementary file 1 — Additional file 1: Figure S1. Correlation between GBP1 expression and infiltrating immune cells in MCP-counter and quanTIseq algorithms. [file 12967_2023_4837_MOESM1_ESM.tif]

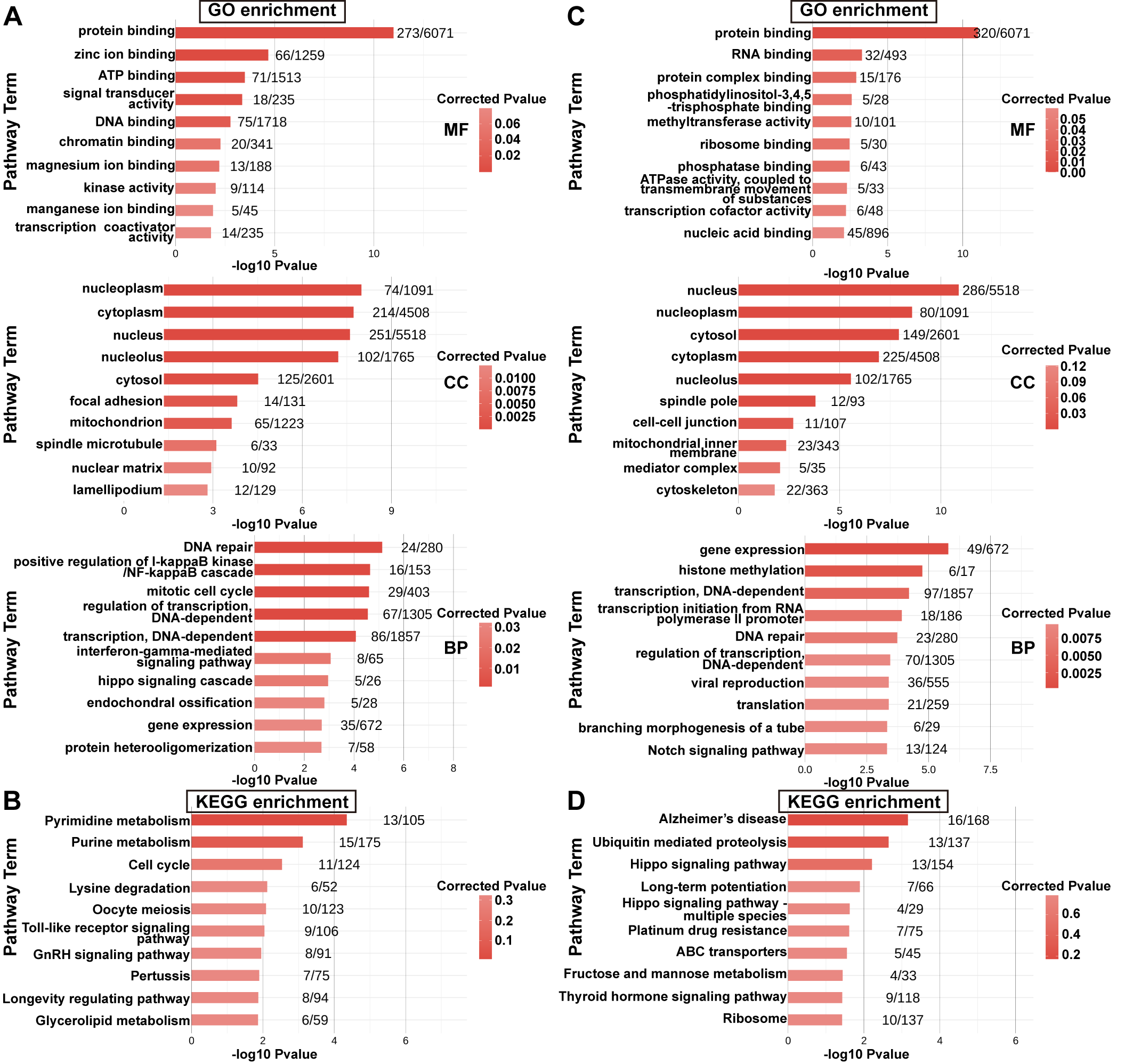

Supplement: Supplementary file 2 — Additional file 2: Figure S2. AS event-related gene enrichment analysis. (A) GO analysis of AS event-related genes after GBP1 overexpression. (B) KEGG analysis of AS event-related genes after GBP1 overexpression. (C) GO analysis of AS event-related genes after GBP1 expression inhibition. (D) KEGG analysis of AS event-related genes after GBP1 expression inhibition. AS: alternative splicing, MF: molecular function, CC: cellular component, BP: biological process. [file 12967_2023_4837_MOESM2_ESM.tif]

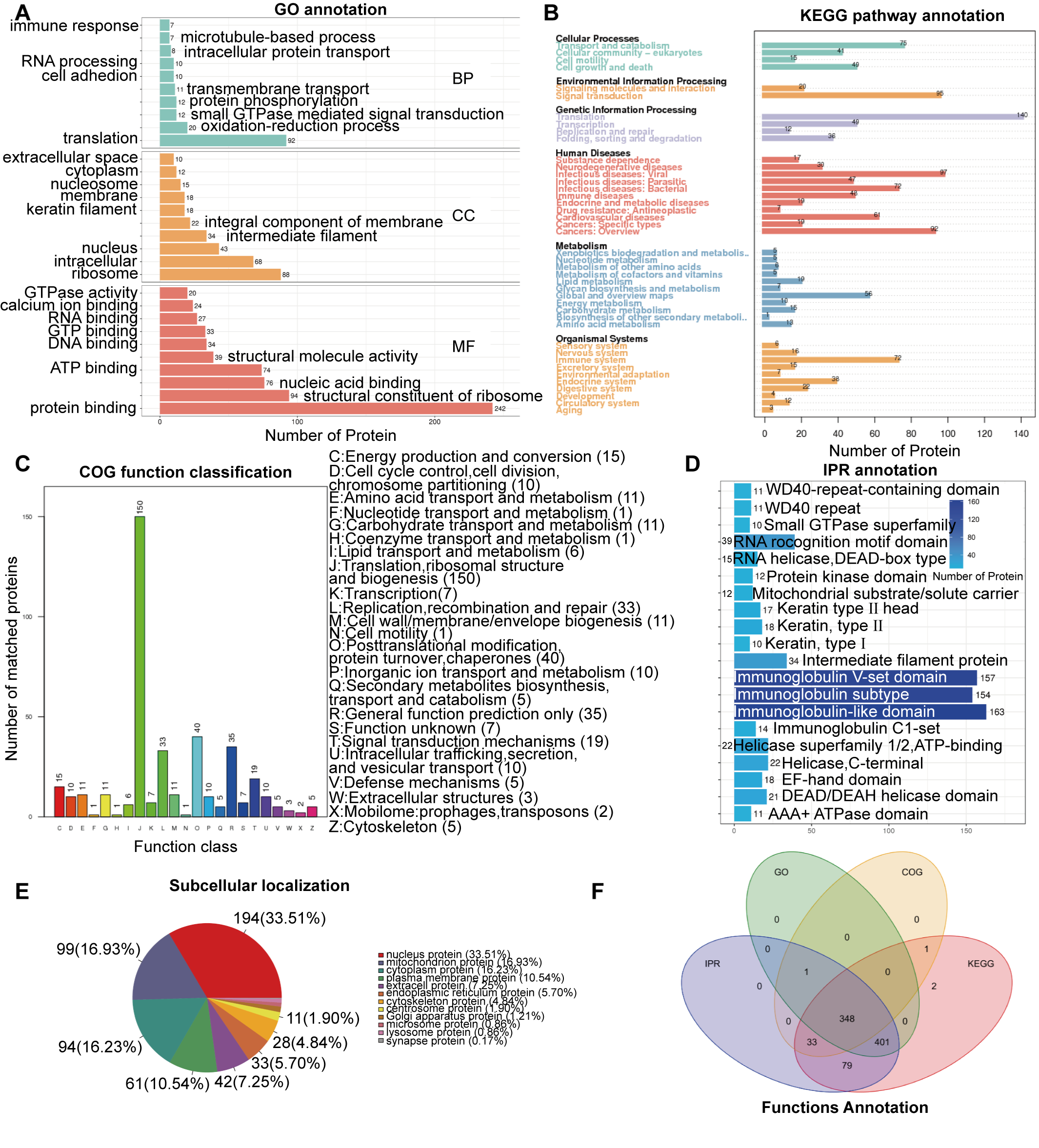

Supplement: Supplementary file 3 — Additional file 3: Figure S3. Enrichment analysis of CoIP-MS original results, including GBP1-IP group detected (not detected in IgG-IP group), IgG-IP group detected (not detected in GBP1-IP group) and two groups detected. (A) GO annotation of interacting proteins in terms of biological processes, cellular components, and molecular functions. (B) KEGG analysis of interacting proteins. (C) COG function classification of interacting protein pathways. (D) IPR analysis of interacting proteins. (E) Subcellular localization of interacting proteins. (F) Venn diagram showed the number of proteins annotated by GO, KEGG, COG and IPR. [file 12967_2023_4837_MOESM3_ESM.tif]
